# Global burden of tobacco-induced atrial fibrillation/flutter: Trends from 1990 to 2021 and projections to 2045 based on the Global Burden of Disease study

**DOI:** 10.18332/tid/216373

**Published:** 2026-02-20

**Authors:** Lei Chen, Zhiqiang Zhang, Litao Wang, Ruimin Pan, Jing Yang, Weihong Lin, Jilang Zeng, Cheng Yu, Lianglong Chen

**Affiliations:** 1 Department of Cardiology, Fujian Medical University Union Hospital, Fujian Cardiovascular Medicine Center, Fujian Institute of Coronary Artery Disease, Fujian Cardiovascular Research Center, Fujian Medical University Heart Center, Fuzhou, China; 2 Department of Cardiology, Tianjin Medical University General Hospital, Tianjin Medical University, Tianjin, China

**Keywords:** atrial fibrillation/flutter, tobacco, Global Burden, sociodemographic index (SDI), DALYs

## Abstract

**INTRODUCTION:**

Tobacco use is a key modifiable risk factor for atrial fibrillation/flutter (AF/AFL). We assessed global, regional, and national burdens attributable to smoking from 1990 to 2021 and projected future trends.

**METHODS:**

Data on deaths and DALYs were extracted from the GBD 2021 study. Main statistical analyses included the calculation of ASMR and ASDR. Trends were quantified using the estimated annual percentage change (EAPC). An age-period-cohort (APC) model was employed to decompose the effects of age, period, and birth cohort. Furthermore, a Bayesian age-period-cohort (BAPC) model was utilized to project the disease burden through 2045.

**RESULTS:**

In 2021, AF/AFL related to tobacco caused around 396000 cases, more than 10000 deaths, and 400000 disability-adjusted life years (DALYs). Compared with 1990, absolute cases and DALYs increased, while ASDR and ASR DALY declined (20%). High SDI regions improved most; low SDI regions lagged. Men and older adults bore disproportionate burdens. By 2045, deaths are projected to reach 14500 and 4200, respectively. Despite rising absolute DALYs, ASMR and ASDR are expected to decline in both sexes.

**CONCLUSIONS:**

Although standardized rates declined, the absolute burden of tobacco-related AF/AFL continues to rise, highlighting the urgent need for stronger tobacco control and targeted interventions in vulnerable populations.

## INTRODUCTION

Atrial fibrillation (AF) and atrial flutter (AFL) are major contributors to the global burden of cardiovascular diseases, and as a growing epidemic, their health impacts extend across different socioeconomic groups^1^. According to the Global Burden of Disease (GBD 2021) study, AF/AFL caused approximately 10012.7 deaths worldwide and 101.4 disability-adjusted life years (DALYs) per 100000 population in 2021, making them the fastest-growing sources of health loss related to persistent arrhythmias^2^. There is an urgent need to control modifiable risk factors at an early stage to lower the occurrence and related complications of AF/AFL. Numerous factors have been identified as closely associated with the onset and progression of AF/AFL, including genetic susceptibility, structural cardiac abnormalities, hypertension, obesity, smoking, sleep apnea syndrome, and lifestyle factors^3^. Notably, smoking is recognized as a prominent modifiable behavioral risk factor. Evidence from a 2024 large cohort study showed that smoking ranks as the third major attributable risk for AF/AFL, after hypertension and obesity, contributing to 12.3% of global AF/AFL etiology^4^. Nicotine and tar in tobacco significantly increase the risk of incident AF/AFL and the likelihood of paroxysmal AF/AFL progressing to persistent forms by inducing atrial fibrosis, promoting sympathetic overactivation, and causing electrical remodeling (e.g. slowed conduction velocity, reduced voltage)^5^. Although global smoking prevalence has declined due to public health policies, regional disparities continue to cause uneven distribution of the AF/AFL burden. High sociodemographic index (SDI) regions have significantly reduced smoking-related AF/AFL incidence through strict tobacco control measures; conversely, low- and middle-SDI regions encounter growing AF/AFL and cardiovascular events owing to elevated male smoking rates and inadequate medical resources^6,7^. However, current research has not systematically analyzed the spatiotemporal evolution and regional inequalities of the smoking-related AF/AFL burden^8^.

To address these knowledge gaps, this study systematically evaluated the spatiotemporal evolution and socioeconomic inequalities of the global AF/AFL burden attributable to tobacco exposure from 1990 to 2021. This study systematically assessed the spatiotemporal evolution and socioeconomic disparities of the global AF/AFL burden attributable to tobacco exposure from 1990 to 2021. The goal of this study is to present a thorough worldwide evaluation of the AF/AFL burden that is directly related to tobacco use between 1990 and 2021. We aim to identify high-risk populations and offer a scientific foundation for focused tobacco control and cardiovascular disease prevention strategies by examining spatial-temporal trends across various SDI regions and age groups and projecting future trends to 2045.

## METHODS

### Study design and data sources

In order to investigate heterogeneity in the worldwide burden of smoking-related AF/AFL, this study carried out a secondary analysis of the GBD 2021 dataset, which includes attributable burdens for 87 risk factors between 1990 and 2021, spanning 7 super-regions, 21 geographical areas, 204 countries, and 811 local-level regions. The study data consisted of :1) burden indicators – AF/AFL DALYs and smoking-attributable mortality from 1990 to 2021 at global, regional, and national levels, disaggregated by sex and age group; 2) demographic data – population size and age structure for the same period; 3) social development indices – SDI measures for 204 nations covering 1990–2021; 4) forecast information – population projections for 2022–2050 available in the GBD database; and 5) standardized reference – GBD standard age-structure data. Data were obtained from the Global Health Data Exchange (GHDx), which is managed by the Institute for Health Metrics and Evaluation (IHME) the Guidelines for Accurate and Transparent Health Estimates Reporting (GATHER), ensuring reproducibility and transparency in data modeling, estimation, and validation (Supplementary file Figure SF1). The GBD framework makes use of sophisticated statistical techniques like spatiotemporal Gaussian process regression (ST-GPR), which generates 1000 posterior draws for each quantity of interest to quantify uncertainty, and DisMod-MR 2.1, a Bayesian meta-regression tool, which borrows strength across time, geography, and covariates to address data sparsity and missingness.

### Disease definition and measurement

Atrial fibrillation originates from rapid and disorganized excitation in the atria, presenting as a supraventricular arrhythmia with disordered atrial electrical activity and ineffective mechanical contractions^9^. Atrial flutter is a type of supraventricular tachycardia characterized by atrial reentrant circuits, often involving macro-reentry^10^. The International Classification of Diseases (ICD-9 and ICD-10) was applied to identify AF/AFL. All cardiovascular conditions coded 427.3-427.32 in ICD-9 and I10-I10 in ICD-10 were defined as AF/AF^11^. Cases satisfying ICD criteria and confirmed by ECG were incorporated into the analysis^4,12^.

DALYs quantified the disease burden as the sum of years of life lost (YLL) and years lived with disability (YLD). YLL was derived from the difference between age at death and standard life expectancy, while YLD was calculated using AF/AFL-specific disability weights (e.g. 0.099 for heart failure comorbidity)^13^.

Sociodemographic development was assessed using the SDI, which integrates per capita income, education level, and fertility rate (scale 0–1). Based on quintile thresholds, 204 countries were classified into high, high-middle, middle, low-middle, and low SDI regions to stratify AF/AFL burden by SDI level^2^. The SDI quintiles in our analysis are pre-aggregated categories provided by the GBD database. The assignment of countries to these quintiles is updated annually (1990–2021) according to changes in socioeconomic status, so the composition of each SDI group varies over time and reflects the developmental stage of countries in each specific year.

### Estimation framework

GBD 2021 applied a Comparative Risk Assessment (CRA) framework, using population attributable fraction (PAF) to estimate the burden of smoking-associated AF/AFL. The GBD network integrated all available data on age, time, geography, and diverse health causes within a Bayesian framework to generate disease estimates. The GBD network integrated all available data on age, time, geography, and diverse health causes within a Bayesian framework to generate disease estimates. The study focused on statistical measures such as total deaths, crude mortality rates for all ages, age-standardized mortality rate (ASMR), relative percentage change, and 95% uncertainty intervals (UIs) for these estimates. In this study, results are reported with 95% uncertainty intervals (UIs) rather than standard confidence intervals (CIs). Unlike CIs, which mainly reflect sampling variability, UIs incorporate additional sources of error, including data quality issues, missing data across regions, and modeling assumptions, and therefore more comprehensively represent the precision of our estimates. We use 95% CIs only for the estimated annual percentage change (EAPC). Following GBD methodology, 95% UIs were determined by the 2.5th and 97.5th percentiles of the distribution of 1000 posterior draws. In all relevant figures, these UIs are visualized as error bars or shaded areas. GBD 2021 introduced a new method to address unexplained heterogeneity across different input datasets and to complement conventional relative risk (RR) results, though secondhand smoke exposure was not included.

### Statistical analysis

This study used the GBD CRA framework to systematically evaluate the burden of smokingrelated AF/AFL and its spatiotemporal trends from 1990 to 2021 in 21 regions and 204 countries, stratified by sex, age group, and SDI (comprising 34 High, 44 High-middle, 42 Middle, 48 Low-middle, and 36 Low SDI countries/territories). The trends in the agestandardized DALY rate (ASDR) and agestandardized mortality rate (ASMR) were quantified using estimated annual percentage change (EAPC) and average annual percentage change (AAPC). EAPC was estimated with a loglinear regression model, ln (ASR) = α + β×year + ε, and model fit was assessed by the coefficient of determination (R^2^) and p-values. An age-period-cohort (APC) model was then applied to separate overall trends into age, period, and cohort effects. In order to capture deviations from the overall linear trend (net drift), nonlinear trends (curvatures) were obtained as the second order differences of the age, period, and cohort effects. These nonlinear components allow for the detection of significant variations in risk across ages, periods, and birth cohorts because they are mathematically identifiable and independent of the particular constraints used to solve the APC identifiability problem. Wald χ^2^ tests were used to assess their statistical significance.

A Bayesian age period cohort (BAPC) model was used to forecast the ASDR and ASMR of smoking-related AF/AFL through 2045. Non-overlapping 95% uncertainty intervals (UIs) were used as the criterion for statistical significance in group comparisons by sex and SDI region, and Spearman’s rank correlation was employed to investigate relationships between SDI and disease burden indicators across regions and years.

Model assumptions, together with the linearity of the log rate-time relationship and the presence of homoscedastic residuals, were validated through the use of residual plots. In addition, R^2^ values were tracked globally and regionally as a confirmation of sufficient fitting. Model stability was tested by means of alternative joinpoint models and the main GBD modeling, which involved out-of-sample cross-validation, wherein predictive performance was evaluated through the use of root mean square error (RMSE). All statistical analyses were carried out using R (version 4.5.1), and p<0.05 was regarded as statistically significant.

## RESULTS

### Tobacco impact on atrial fibrillation and flutter across different ages

**Figure 1:**
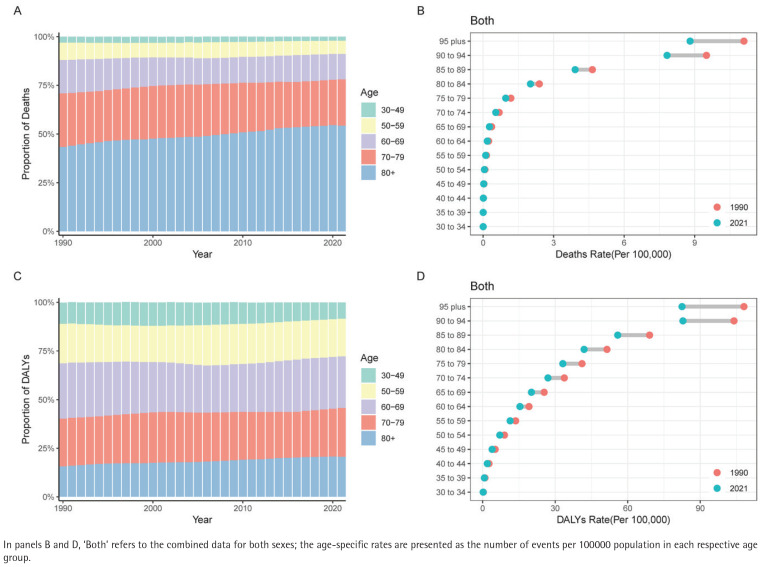
Global age-specific trends in mortality and disability-adjusted life years (DALYs) for atrial fibrillation and atrial flutter (AF/AFL) attributable to tobacco use, 1990–2021: A) Proportion of deaths due to AF/AFL attributable to tobacco by age group; B) Age-specific AF/AFL mortality rates in 1990 versus 2021; C) Proportion of DALYs attributable to AF/AFL by age group; D) Age-specific DALY rates in 1990 vs 2021

The AF/AFL burden showed a clear age shift, with the proportion of deaths among those aged ≥80 years rising significantly from about 45% in 1990 to over 55% in 2021, an increase of 10%. Meanwhile, the age group of 70–79 years decreased from about 25% to 20%, the age group of 60–69 years from 18% to 15%, the age group of 50–59 years from 10% to 8%, and the age group of 30–49 years from 2% to 1%. All age groups showed lower mortality rates and DALYs rates in 2021 compared with 1990. Notably, the mortality rate in the ≥95 years group decreased the most significantly, from about 10 per 100000 to 8 per 100000, a drop of 20%. In the age group of 75–94 years, the DALY rate fell from 80–90 per 100000 to 60–70 per 100000 (Figure 1).

However, these indicators still show a significant increase with age, with the mortality and DALYs rates for those aged ≥95 years remaining the highest, reaching approximately 10–11 per 100000 and 90–100 per 100000, respectively. The proportion of the population aged ≥80 years in DALYs increased from 18% in 1990 to 20% in 2021. The age group of 70–79 years remained stable at 23%, while the age group of 60–69 years remained stable 30% with little change over 31 years. The 50–59 years age group dropped from about 20% to 18%, and the 30–49 years age group decreased from 9% to 8%. Middleaged individuals between 50–69 years accounted for a total of 48% of the DALY burden. Although DALY rates declined across all age groups, older age groups continued to have much higher rates than those for younger groups. In those aged ≥90 years, the rate was still 70–80 per 100000 (Figure 1).

### Tobacco impact on atrial fibrillation and flutter across sex

According to mortality proportion data, the percentage of men aged ≥80 years increased from 40% in 1990 to 50% in 2021, while the proportion of women in the same age group rose from 55% to 65%, consistently remaining 15% higher than that of men. At the same time, the proportion of men aged 60–69 years decreased from 20% to 15%, while the age group of 70–79 years remained stable around 25%, while for women in the age group of 60–69 years dropped from 15% to 10% and the 70–79 years age group from 25% to 20%. Notably, the proportion of men aged ≥80 years grew more rapidly during the years 2005 to 2010, while women showed a more stable upward trend. In 2021, the mortality rate for males aged ≥95 years was 17 per 100000, and among the age group of 90–94 years, it was 15 per 100000, much higher than the 5.5 and 4.5 per 100000 observed in women of the same age.

From 1990 to 2021, the decline in mortality among elderly men aged 85–95 years ranged from 13% to 15%, while the reduction in women of the same ages was between 23% and 34%, significantly higher than that of men (Supplementary file Figure SF2). In 2021, the ASMR in global males was 0.2 per 100000 (95% UI: 0.1–0.3) compared with 0.1 per 100000 (95% UI: 0.0–0.1) in females (Table 1).

**Table 1:** Global mortality burden and temporal trends of atrial fibrillation and atrial flutter (AF/AFL) attributable to tobacco use from 1990 to 2021, by sex and sociodemographic index (SDI)

	*Mortality (95% UI)*		*All-age mortality rate/100000 (95% UI)*		*Age-standardized mortality rate/100000 (95% UI)*		*EAPC (95% CI)*
	*2021*	*Change 1990–2021*	*2021*	*Percent change 1990–2021*	*2021*	*Percent change 1990–2021*	*Net drift of mortality*
**Global**	10012.7 (5851.5–14648.8)	109.94 (81.91–142.21)	0.1 (0.1–0.2)	41.89 (22.95– 63.71)	0.1 (0.1–0.2)	-19.11 (-29.39 – -7.3)	-0.86 (-1.05 – -0.66)
**Sex**							
Male	7304.5 (4319.9–10551.7)	121.18 (86.19–167.58)	0.2 (0.1–0.3)	50.03 (26.3– 81.5)	0.2 (0.1–0.3)	-17.85 (-29.45 – -1.25)	-0.8 (-1.02 – -0.59)
Female	2708.2 (1520.2–4079.1)	84.63 (55.61–118.5)	0.1 (0–0.1)	24.34 (4.79–47.14)	0.1 (0–0.1)	-30.37 (-40.81– -18.09)	-1.2 (-1.67 – -0.73)
**SDI**							
High	3200.9 (1848.4–4790.3)	60.32 (46.92–74.3)	0.3 (0.2–0.4)	28.89 (18.11–40.12)	0.1 (0.1–0.2)	-28.07 (-32.88 – -22.89)	-1.04 (-1.45 – -0.62)
High-middle	2367 (1344–3493.1)	121.27 (77.63–174.43)	0.2 (0.1–0.3)	80.46 (44.87–123.81)	0.1 (0.1–0.2)	-9.22 (-26.6–12.61)	-0.49 (-0.93 – -0.04)
Middle	2723.5 (1588.4–4034.1)	152.47 (100.74 –238.5)	0.1 (0.1–0.2)	77.65 (41.25–138.19)	0.1 (0.1–0.2)	-24.57 (-39.36 – -1.07)	-1.12 (-1.47 – -0.77)
Low-middle	1426 (843–2092.9)	186.93 (127.09– 287.64)	0.1 (0–0.1)	73.46 (37.28–134.35)	0.1 (0.1–0.2)	8.68 (-13.56–46.97)	-0.03 (-0.52–0.46)
Low	285.2 (151.6–457.5)	136.28 (90.42–219.26)	0 (0–0)	6.01 (-14.57– 43.23)	0.1 (0–0.1)	1.34 (-18.35–39.35)	-0.38 (-1.3– 0.55)

EAPC: estimated annual percentage change. UI: uncertainty interval. CI: confidence interval.

The DALYs proportions in men show that the 60–69 years age group held the leading share throughout the period, increasing from 30% in 1990 to 35% in 2021, with a marked increase especially after 2010. Meanwhile, the proportion of the 50–59 years age group declined from 25% to 20%, while the proportion for those aged ≥80 years increased from about 12% to around 15% (Supplementary file Figure SF3). In 2021, among the 396000 global cases, 80.4% were observed in men, with high and middle SDI regions contributing 58.3% of the burden. In 2021, male DALYs stood at 8.0 per 100000 population (95% UI: 4.7–11.9), compared to 2.0 per 100000 population (95% UI: 1.1–3.0) for females. Over time, the relative decline was greater for females, with a 30.5% decrease in female ASDR compared to a 19.2% decrease in male ASDR (Table 2).

**Table 2:** Global disability-adjusted life years (DALYs) burden and temporal trends of atrial fibrillation and atrial flutter (AF/AFL) attributable to tobacco use from 1990 to 2021, by sex and sociodemographic index (SDI)

	*DALYs (95% UI)*		*All-age DALYs rate/100000 (95% UI)*		*Age-standardized DALYs rate/100000 (95% UI)*		*EAPC (95% CI)*
	*2021*	*Change 1990–2021*	*2021*	*Percent change 1990–2021*	*2021*	*Percent change 1990–2021*	*Net drift of DALYs*
**Global**	396210.4 (232053.5–586951.5)	81.15 (68.17–94.79)	5 (2.9–7.4)	22.43 (13.66–31.66)	4.6 (2.7–6.9)	-19.99 (-25.23 – -14.15)	-0.78 (-0.81– -0.74)
**Sex**							
Male	318498.3 (186931.5–469855)	87.08 (72.91–104.22)	8 (4.7–11.9)	26.9 (17.29–38.52)	8.1 (4.7–12)	-19.18 (-25.2 – -11.99)	-0.73 (-0.76 – -0.69)
Female	77712.2 (44155.2–118402.2)	60.31 (46.59–76.33)	2 (1.1–3)	7.96 (-1.28–18.74)	1.7 (0.9–2.5)	-30.5 (-36.5 – -23.85)	-1.22 (-1.28 – -1.15)
**SDI**							
High	114874.8 (66835.8–172129.4)	39.72 (28.84–48.66)	10.5 (6.1–15.7)	12.33 (3.58–19.51)	5.6 (3.3–8.4)	-24.99 (-30.07 – -20.72)	-1.01 (-1.05 – -0.97)
High-middle	99329.2 (57532.5–147759.8)	86.56 (69.66–105.96)	7.6 (4.4–11.3)	52.15 (38.37–67.97)	5 (2.9–7.5)	-9.01 (-17.04–0.23)	-0.25 (-0.31 – -0.19)
Middle	116419.4 (66895.4–173374.1)	122.66 (98.58–153.54)	4.8 (2.7–7.1)	56.67 (39.73– 78.4)	4.5 (2.6–6.6)	-20.44 (-29.16 – -9.04)	-0.78 (-0.84 – -0.71)
Low-middle	53909.2 (30795–79712.4)	116.06 (93.74–143.29)	2.8 (1.6–4.1)	30.62 (17.13–47.08)	4.1 (2.4–6)	-10.61 (-19.78–1.09)	-0.45 (-0.51 – -0.39)
Low	11279.6 (6369.1–17287.6)	96.43 (74.82–124.03)	1 (0.6–1.5)	-11.88 (-21.57–0.51)	2.5 (1.4–3.9)	-12.99 (-22.8–0.54)	-0.5 (-0.63 – -0.36)

EAPC: estimated annual percentage change. UI: uncertainty interval. CI: confidence interval.

### Tobacco impact on atrial fibrillation and flutter across different SDI levels

Age patterns displayed marked differences across regions. In high-SDI regions, mortality shifted to older populations, with deaths among those aged ≥80 years rising from 50% in 1990 to 60% in 2021. In contrast, the same age group accounted for only 30% to 40% of deaths in low SDI regions, where younger groups contributed proportionally more. For example, in 2021, people in the age group of 50–59 years still represented around 15% of deaths in low-SDI settings (Supplementary file Figure SF4). The DALYs burden showed a different age pattern from mortality. In high-SDI regions, 30% of DALYs were concentrated in the age group of 60–69 years, while in low-SDI areas, 40% occurred in ages 30–59 years. The DALY rate among people aged 65–69 years declined by 40% in high-SDI regions but only 20% in low-SDI regions. For those ≥95 years, rates decreased 17% in high-SDI regions but rose 5% in middle/low-SDI settings (Supplementary file Figure SF5).

Deaths in high-SDI regions reached 3200.9 (95% UI: 1848.4–4790.3) in 2021, a 60% rise from 1990, while high-middle and middle-SDI regions experienced increases of 121% and over 100%, respectively, despite moderate ASMR reductions. Low and low-middle SDI regions saw the steepest increases, up to 187% (Table 1).

### Age-period-cohort analysis on disease burden

Local drift was negative (-0.86%/year). Women improved faster (-1.22%) than men (-0.73%). DALYs present a ‘U-shaped’ curve with 65 years as the turning point, and women aged 30–39 years had positive drift (+1.0%). Age effects indicated an exponential rise in risk, 8.5 times higher in those >80 years than within the age range of 60–64 years; gender differences peak in the 50–69 years working age group (DALYs, male:female=2.8:1). Period effects revealed accelerated declines followed by rebounds after 2010; 2005 was a key turning point, with male mortality increasing by 0.9% and female improvements (33%) exceeding those of males (14%). Cohort analysis showed decreasing risks for those born before 1940, while the risk for cohorts born after the 1980s reverses (RR≈1.10), with a more significant increase in women (Supplementary file Figure SF6).

### Global trends in atrial fibrillation and flutter caused by tobacco

In 2021, 10012.7 (95% UI: 55851.5–14648.8) deaths related to tobacco-associated AF/AFL were recorded globally, and the burden of tobacco-related AF/AFL diseases showed an overall declining trend from 1990 to 2021, with a 19.1% (95% UI: -29.4 – -7.3) decrease in mortality, while the number of deaths increased by 109.9% (Table 1).

In South Asia, the number of deaths in India increased from 285000 in 1990 to 943000 in 2019, with DALYs rising from 16 million to 34.8 million. In Bangladesh, the number of deaths nearly quadrupled, increasing from 65000 to 248000, while DALYs rose from 2.4 million to 7.51 million. In contrast, the situation in high SDI regions is more optimistic; Japan’s ASMR declined by an average of -3.5% per year, with deaths rising slightly from 255000 to 341000, but total DALYs experienced a minor decrease. The advancement in Eastern Europe has been comparatively limited. Deaths in Russia increased from 118000 to 244000, and DALYs rose from 7.8 million to 13 million (Supplementary file Table ST1).

**Figure 2:**
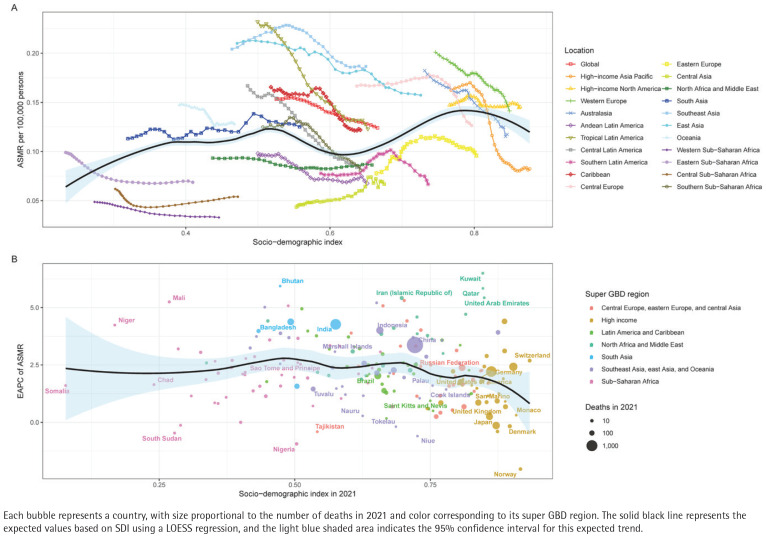
Correlation between sociodemographic index (SDI) and atrial fibrillation and atrial flutter (AF/AFL) mortality attributable to tobacco use across 204 countries and territories, 1990–2021: A) Age-standardized mortality rate (ASMR) of AF/AFL attributable to tobacco across global regions in relation to SDI from 1990 to 2021; B) Estimated annual percentage change (EAPC) in ASMR vs SDI in 2021 by country

The global average curve (Figure 2, black line) shows that the mortality rate is lowest in regions with a medium SDI (0.5–0.6), while high and low SDI regions show higher levels. The medium SDI areas in South Asia and East Asia achieved a peak (around 0.22 per 100000), significantly higher than the global average level (0.15 per 100000). In North Africa and the Middle East, mortality rose steadily with SDI, rising from 0.09 to 0.20 per 100000 with higher SDI levels. Sub-Saharan Africa showed strong heterogeneity, with heavier burdens in the east and south, while the central and western regions are lighter (Figure 2).

**Figure 3:**
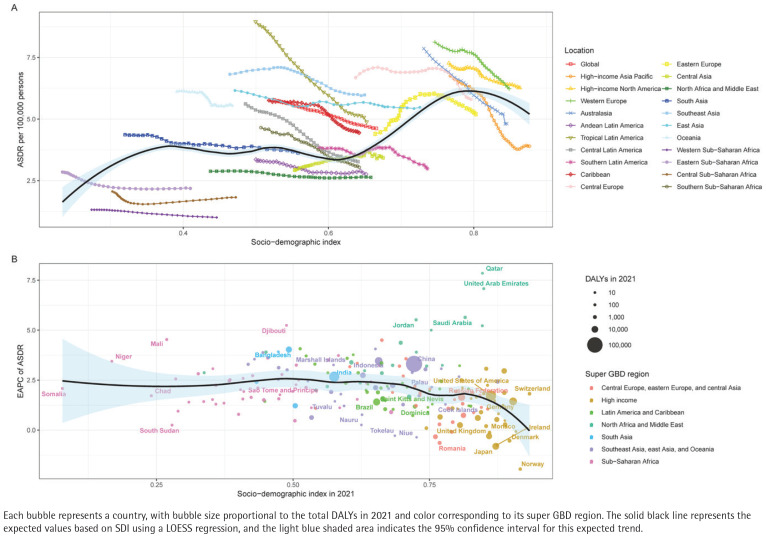
Correlation between sociodemographic Index (SDI) and atrial fibrillation and atrial flutter (AF/AFL) disability-adjusted life years (DALYs) attributable to tobacco use across 204 countries and territories, 1990–2021: A) Age-standardized DALY rate (ASDR) of AF/AFL attributable to tobacco across global regions in relation to SDI from 1990 to 2021; B) Estimated annual percentage change (EAPC) in ASDR vs SDI in 2021 by country

In contrast, Western Europe sustains the lowest ASDR, while Eastern Europe and Central Asia carry the highest burdens. Although most high SDI regions achieve modest declines (EAPC= -1.2%), many low SDI countries improve slowly, and some Middle Eastern and populous nations, notably China and India, combine high absolute burdens with persistently positive EAPCs, underscoring global inequities in tobacco control and AF/AFL prevention (Figure 3).

### Temporal trends in atrial fibrillation and flutter caused by tobacco

From 1990 to 2021, tobacco-attributable AF/AFL burden declined in stages. ASDR decreased steadily, with five joinpoints observed and a global AAPC of -0.72% (Supplementary file Table ST2). ASMR declined by an average of 0.66% per year, with turning points in 1996, 2003, and 2006. The overall annual average decline in ASDR burden is 0.72%, particularly reaching the fastest decline rate (-1.11%) from 2011 to 2014, after which it entered a relatively moderate period (-0.68%) (Supplementary file Figure SF7).

The male ASMR burden has two turning points, with the APC accelerating from -0.01% to -1.00%, and the decline rate slows after 2006. Females have five turning points, with a significant decline from 2003 to 2013, and the overall improvement is greater than that of males. The male ASDR burden showed a turning point in 2007, with the APC accelerating from -0.85% to -0.90%, and the fastest decline occurred from 2010 to 2014. The female ASDR burden experienced the most significant improvement from 2004 to 2013, followed by a slight slowing of the decline rate (Supplementary file Figure SF8).

### Atrial fibrillation and flutter burden from tobacco and 2045 forecast

**Figure 4:**
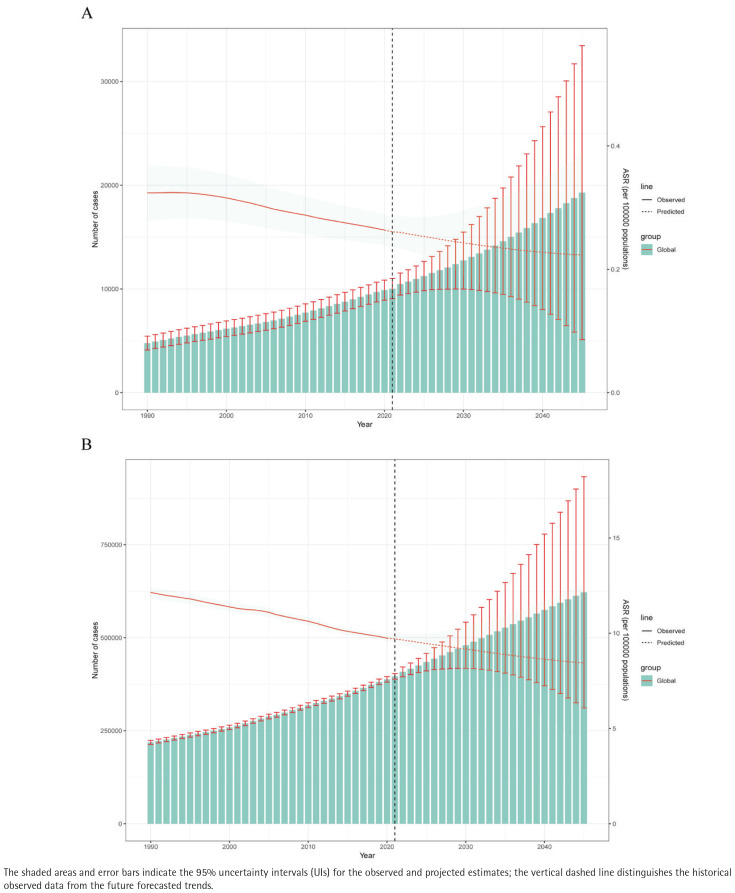
Temporal trends and future projections of the burden of atrial fibrillation and atrial flutter (AF/AFL) attributable to tobacco use at the global level, 1990–2049: A) Observed and projected total number of deaths (represented by bars, left axis) and age-standardized mortality rate (ASMR; represented by lines, right axis) of AF/AFL; B) Observed and projected total number of disability-adjusted life years (DALYs; represented by bars, left axis) and age-standardized DALY rate (ASDR; represented by lines, right axis) of AF/AFL

AF/AFL mortality rose from 4000 in 1990 to 11000 in 2020, with projections reaching 19000 by 2045. The ASMR declined from 0.35 per 100000 in 1990 to 0.25 per 100000 in 2020 and is projected at 0.22/100000 in 2045 (-12%) (Figure 4).

The DALYs increased from 215000 cases in 1990 to 400000 cases in 2020 and are projected to reach 625000 cases by 2045, reflecting a growth of 56%. The ADSR decreased from approximately 12 per 100000 in 1990 to about 9 per 100000 in 2020 and is projected to remain at around 8.5 per 100000 by 2045 (Figure 4).

From 1990 to 2020, male death cases increased from 3500 to 7000, while female deaths rose from 1500 to 2500, with projections that by 2045, these numbers will reach 14500 and 4200. During the same period, male DALYs increased from 250000 to 400000, while female DALYs rose from 50000 to 75000. However, male ASMR decreased from 0.55 to 0.45 per 100000, an 18% drop, while female ASMR decreased from 0.18 to 0.12 per 100000, a 33% drop. Male ASDR fell from 22 to 15 per 100000, a 32% decrease, while female ASDR dropped from 7 to 4 per 100000, a 43% decrease (Supplementary file Figure SF9).

## DISCUSSION

This study provides a comprehensive and up-to-date assessment of the global burden of AF/AFL attributable to tobacco use using the GBD 2021 dataset. The primary findings are as follows. First, in 2021, tobacco use accounted for approximately 10012.7 deaths and 396210.4 DALYs due to AF/AFL globally. Second, while the age-standardized mortality and DALY rates showed a declining trend from 1990 to 2021, the absolute numbers of deaths and DALYs increased by 109.94% and 81.15%, reflecting the impact of population growth and aging. Third, we observed significant sex and regional disparities, with males and individuals in low SDI regions experiencing a disproportionately higher disease burden. These results underscore the persistent challenge of tobacco use as a major modifiable risk factor for AF/AFL, particularly in the context of an aging global population.

Tobacco has become a major risk factor for the global burden of AF/AFL, promoting the initiation and progression of AF/AFL through multiple pathways^14^. Nicotine-driven receptor activation and carbon monoxide-induced hypoxia lead to electrophysiological instability and increased hemodynamic load^15^. Oxidative stress induced by free radicals from smoke, along with chronic inflammation mediated by NF-κB/NLRP3, results in endothelial dysfunction, advances atherosclerosis, and establishes a hypercoagulable state^16,17^. The RAAS and TGF-β/Smad pathways trigger myocardial fibrosis and remodeling, increasing the burden of arrhythmias (especially atrial fibrillation), heart failure, and stroke risk^18^. Smoking cessation can lower risks via various pathways, reinforcing the need to integrate systematic smoking cessation interventions into the comprehensive care of cardiovascular and atrial fibrillation patients to enhance long-term survival^19^.

Although smoking-related AF/AFL mortality and incidence rates have declined over the past 30 years, the global number of cases among smokers is still rising, suggesting that population aging and population growth have offset some of the benefits brought by tobacco control policies^20^. Therefore, it is necessary to conduct in-depth research on the current status and evolving trends of AF/AFL attributable to tobacco and guide the development of effective public prevention strategies to reduce the burden.

Based on GBD 2021 data, this study comprehensively analyzed global trends of smoking-related AF/AFL from 1990 to 2021 and quantified the disease burden. Stratified assessments by sex, age group, and SDI level were performed to characterize the distribution of burden and to identify high-risk populations. We further focused on regional heterogeneity across 204 countries and predicted disease burden evolution trends through 2045. Using DALYs as a core indicator, we provide evidence for the epidemiological study of tobacco-related arrhythmias.

Based on GBD 2021 data, this study comprehensively analyzed the global trends of tobacco-related AF/AFL from 1990 to 2021 and quantified the disease burden. By stratifying the analysis by sex, age group, and SDI, the characteristics of high-risk populations were systematically analyzed. Further focus on the regional heterogeneity of 204 countries, we predict the trends of disease burden evolution up to 2045. Using DALYs as the core indicator, the study provides an evidence-based foundation for epidemiological research on tobacco-induced arrhythmias.

This study shows that the burden of tobacco-related AF/AFL exhibits a significant age effect. The proportion of deaths in the population aged ≥80 years increased from 45% in 1990 to 55% in 2021, and the proportion of DALYs also rose from 18% to 20%, while the mortality and DALYs rate for those aged ≥95 years have always been the highest. This trend of concentration suggests the synergistic effect of aging and tobacco exposure. Aging leads to atrial fibrosis, electrophysiological remodeling, and decreased cardiac reserve, making older hearts more susceptible to smoking damage^21^. Additionally, older people have greater cumulative tobacco exposure and commonly have risk factors such as hypertension, coronary heart disease, and diabetes, further amplifying AF/AFL risk^22^. Notably, the mortality rate among people aged ≥95 years decreased by 20% in high SDI regions, indicating that medical interventions and tobacco control measures can alleviate some of the burden. However, the same age group in low SDI regions showed an increase, suggesting that regional disparities may continue to widen in the context of population aging^23^.

The burden of tobacco-related AF/AFL is significantly higher in men than in women. In 2021, the incidence rate in men was approximately 4 times that of women, and the mortality rate was about 2 times higher, with the disparity particularly pronounced in the group aged ≥95 years. Mechanistically, men have higher smoking rates, start smoking at an earlier age, have greater cumulative exposure, and have lower quit rates^24^. Additionally, men often have other risk factors such as alcohol consumption and metabolic syndrome, which work synergistically with smoking^25-27^. Women benefit from the protective effects of estrogen, resulting in relatively less atrial fibrosis and inflammation, leading to a lower risk level^28^. Notably, female populations show faster improvement under tobacco control policies, with more significant declines in mortality and DALYs rates among older age groups^20^. This suggests that smoking control interventions are more effective for women, but it is concerning that in certain middle SDI countries, the increasing smoking rates among young women are causing a reversal in AF/AFL risk^29^.

The burden of tobacco-related AF/AFL exhibits a significant socioeconomic gradient^30^. In high SDI regions, the burden mainly affects older populations, while low SDI regions show a ‘younger’ trend. In terms of improvement, high SDI regions have greater improvement than low SDI regions. At the national level, China has the heaviest absolute burden, India shows limited improvement, and some high SDI countries in the Middle East, like Qatar and the UAE, and low SDI countries in Sub-Saharan Africa, like Mali and Niger, are showing rapidly worsening trends^31^. Overall, global tobacco control effectiveness is most significant in high SDI regions, while low-middle SDI regions not only lag in improvement but also show an increasing burden among younger people^32^.

The trends revealed by this study indicate that smoking-related AF/AFL is evolving from a controllable disease primarily dependent on lifestyle interventions into a structural public health challenge within the context of aging^33^. If more targeted measures are not taken, future global disease burden gaps may become further entrenched or even widen. The decline in the ASR of global tobacco-related AF/AFL demonstrates that existing interventions have played an important role in reducing relative risk^34^. However, the absolute number of cases and deaths continues to rise, with the disease burden increasingly concentrated among older people. Male improvement lags behind, and low SDI regions even show younger burden patterns. These trends suggest that an aging population, long-term exposure, and regional development imbalances are reshaping the patterns of disease.

Based on the results of this study and related predictive research, future global smoking rates are projected to continue to decline, but the absolute number of tobacco-related deaths and DALYs may continue to increase due to population growth and aging^35^. This trend suggests that tobacco-related AF/AFL will remain an important public health challenge globally for a considerable period.

### Strengths and limitations

Our study has a number of strengths. First, it makes use of the most recent data from the GBD study, which guarantees that the results represent the most recent epidemiological environment and take advantage of the GBD’s consistent data processing and estimation techniques. Second, this research includes future projections, which can help policymakers anticipate future healthcare needs and assess the possible effects of current intervention strategies, in contrast to many earlier studies that only concentrate on historical trends. A more nuanced understanding of the variations in AF/AFL burden is also made possible by the wide geographical scope and the thorough breakdown by age, sex, and region.

There are a number of limitations to this study. First, there may be uncertainty and regional heterogeneity due to regional variations in the quality and completeness of the GBD source data, especially in low and middle SDI areas. Second, bias may result from long-term changes in coding practices, modeling assumptions, and data gaps that affect trend estimates derived from models like the EAPC. Third, the results should be interpreted cautiously because the correlation analyses only show statistical associations rather than causal relationships. Fourth, even after controlling for age and sex, residual confounding from things like food, exercise, and alcohol consumption might still exist. Fifth, the potential for systematic bias brought on by temporal shifts in diagnostic techniques, reporting systems, and coverage is only partially mitigated by coding mapping, automatic speech recognition, and year-based adjustments. Sixth, as previously mentioned, this analysis did not account for exposure to secondhand smoke. Secondhand smoke was excluded due to data constraints, which could have led to residual confounding and understated the complete tobacco-related health effects. Given that it is a known risk factor for respiratory and cardiovascular conditions as well as a number of cancers, our results probably provide a conservative estimate of the overall tobacco-attributable burden, possibly underestimating the health effects on vulnerable groups and non-smokers. Furthermore, the estimates for 2045 are based on past patterns and do not take into consideration upcoming policy modifications, advancements in medicine, or unanticipated worldwide health emergencies. Due to multiple comparisons, a high number of statistical tests raises the possibility of type I errors. The analysis may understate the effects of e-cigarettes and heated tobacco products because it concentrates on conventional tobacco. Lastly, the observed associations may not apply at the individual level and should not be interpreted as direct causal effects because this is an ecological, population-level study.

## CONCLUSIONS

Over the past thirty years, the incidence and mortality rates of tobacco-related have atrial fibrillation and atrial flutter shown an overall declining trend. It is necessary to develop more targeted strategies while continuing universal tobacco control efforts.

## Supplementary Material



## Data Availability

The data supporting this research are available through the Global Health Data Exchange (GHDx): https://vizhub.healthdata.org
