# Supplementary material for: Global burden of tobacco-induced atrial fibrillation/flutter: Trends from 1990 to 2021 and projections to 2045 based on the Global Burden of Disease study
Source: Tob Induc Dis. 2026 Feb 20;24:10.18332/tid/216373. doi: 10.18332/tid/216373 (PMC12937991; doi:10.18332/tid/216373)
Supplement: Supplementary file 1 [file TID-24-29-s1.pdf]

Supplemental Material

Content of Supplemental Material

Supplemental Figures

Figure S1. Methodological framework for estimating the disease burden of atrial fibrillation and flutter. The flowchart illustrates two main analytical pathways: (1) overall disease burden estimation using standardized data, ICD mapping, and cause-of-death databases to calculate DALYs (orange boxes), and (2) smoking-attributable burden assessment through prevalence data extraction, exposure modeling, and population attributable fraction (PAF) calculations (blue boxes). EMR: excess mortality rate; CSMR: cause-specific mortality rate; YLD: years lived with disability; YLL: years of life lost; ST-GPR: spatiotemporal Gaussian process regression.

Figure S2. The temporal change in the sex specific age distribution and death rates of AF/AFL attributable to tobacco globally from 1990 to 2021. (A) Age specific proportion of AF/AFL deaths for males. (B) Comparison of AF/AFL death rates (per 100,000) for males from 1990 to 2021. (C) Age-specific proportion of AF/AFL deaths for females. (D) Comparison of AF/AFL death rates (per 100,000) for females from 1990 to 2021.

Figure S3. The temporal change in the sex specific age distribution and DALYs rates of AF/AFL attributable to tobacco globally from 1990 to 2021. (A) Age-specific proportion of AF/AFL DALYs for males. (B) Comparison of DALYs rates (per 100,000) for males in 1990 vs 2021. (C) Age specific proportion of AF/AFL DALYs for females. (D) Comparison of DALYs rates (per 100,000) for females in 1990 vs 2021.

Figure S4. The temporal change in the age distribution and death rates of AF/AFL attributable to tobacco across five SDI regions globally from 1990 to 2021. (A, B) High SDI: Age-specific proportion of AF/AFL deaths (A) and comparison of AF/AFL death rates (per 100,000) in 1990 vs 2021 (B). Comparison of AF/AFL death rates (per 100,000) for different age groups in the high SDI region between 1990 and 2021. (C) Age-specific proportion of AF/AFL deaths attributable to tobacco in the high-middle SDI region from 1990 to 2021. (D). Comparison of AF/AFL death rates (per 100,000) for different age groups in the high-middle SDI region between 1990 and 2021. (E) Age specific proportion of AF/AFL deaths attributable to tobacco in the middle SDI region from 1990 to 2021. (F) Comparison of AF/AFL death rates (per 100,000) for different age groups in the middle SDI region between 1990 and 2021. (G) Age specific proportion of AF/AFL deaths attributable to tobacco in the low-middle SDI region from 1990 to 2021. (H) Comparison of AF/AFL death rates (per 100,000) for different age groups in the low-middle SDI region between 1990 and 2021. (I) Age specific proportion of AF/AFL deaths attributable to tobacco in the low SDI region from 1990 to 2021. (J). Comparison of

AF/AFL death rates (per 100,000) for different age groups in the low-SDI region between 1990 and 2021.

Figure S5. The temporal change in the age distribution and DALYs rates of AF/AFL attributable to tobacco across five SDI regions globally from 1990 to 2021. (A) Age specific proportion of AF/AFL DALYs in the high SDI region. (B) Comparison of AF/AFL DALYs rates (per 100,000) in the high SDI region between 1990 and 2021. (C) Agespecific proportion of AF/AFL DALYs in the high-middle SDI region. (D) Comparison of AF/AFL DALYs rates (per 100,000) in the high-middle SDI region between 1990 and 2021. (E) Age specific proportion of AF/AFL DALYs in the middle SDI region. (F) Comparison of AF/AFL DALYs rates (per 100,000) in the middle SDI region between 1990 and 2021. (G) Age specific proportion of AF/AFL DALYs in the low-middle SDI region. (H) Comparison of AF/AFL DALYs rates (per 100,000) in the low-middle SDI region between 1990 and 2021. (I) Age-specific proportion of AF/AFL DALYs in the low SDI region. (J) Comparison of AF/AFL DALYs rates (per 100,000) in the low SDI region between 1990 and 2021.

Figure S6. Age-Period-Cohort analysis of AF/AFL mortality and DALYs attributable to tobacco use globally, 1990–2021. (A) Local drift analysis of AF/AFL mortality. (B) Age effects on AF/AFL mortality. (C) Period effects on AF/AFL mortality. (D) Cohort effects on AF/AFL mortality. (E) Local drift analysis of AF/AFL DALYs. (F) Age effects on AF/AFL DALYs. (G) Period effects on AF/AFL DALYs. (H) Cohort effects on AF/AFL DALYs. The vertical error bars indicate the 95% CIs for the estimated rates and rate ratios.

Figure S7. Joinpoint regression analysis of age-standardized rates of AF/AFL attributable to tobacco globally from 1990 to 2021. (A) ASMR of AF/AFL attributable to tobacco with joinpoint regression and estimated APC during different periods. (B) ASDR of AF/AFL attributable to tobacco with joinpoint regression and estimated APC during different periods.

Figure S8. Joinpoint regression analysis of age-standardized death and DALYs rates of AF/AFL attributable to tobacco stratified by sex at the global level, 1990–2021. (A) ASMR of AF/AFL attributable to tobacco in males with joinpoint regression and APC in different periods. (B) ASMR of AF/AFL attributable to tobacco in females with joinpoint regression and APC in different periods. (C) ASDR of AF/AFL attributable to tobacco in males with joinpoint regression and APC in different periods. (D) ASDR of AF/AFL attributable to tobacco in females with joinpoint regression and APC in different periods.

Figure S9. Observed and forecasted numbers and ASR of AF/AFL attributable to tobacco, stratified by sex at the global level, 1990–2049. (A) Observed and projected deaths (bars, left axis) and ASMR (lines, right axis) of AF/AFL attributable to tobacco in males, with 95% uncertainty intervals. (B) Observed and projected deaths (bars, left axis) and ASMR (lines, right axis) of AF/AFL attributable to tobacco in females, with 95% uncertainty intervals. (C)

Observed and projected DALYs (bars, left axis) and ASDR (lines, right axis) of AF/AFL attributable to tobacco in males, with 95% uncertainty intervals. (D) Observed and projected DALYs (bars, left axis) and ASDR (lines, right axis) of AF/AFL attributable to tobacco in females, with 95% uncertainty intervals.

Supplemental Tables

Table S1. Deaths, ASMR, DALYs, ASDR, and EAPCs of AF/AFL attributable to tobacco in 204 countries and territories, 1990–2021.

Table S2. Joinpoint regression analysis of ASMR and ASDR of AF/AFL attributable to tobacco at the global level, 1990–2021.

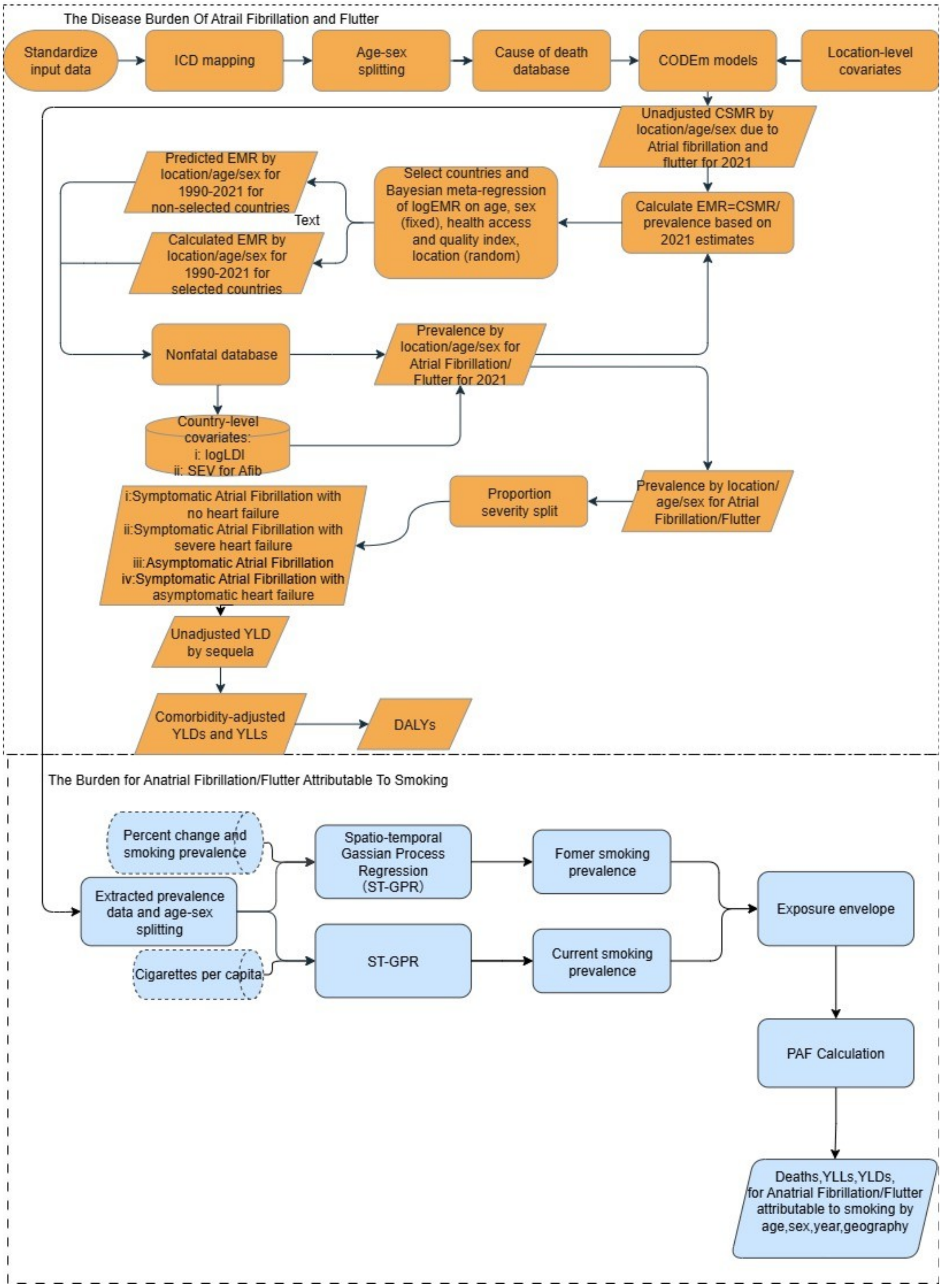

Figure S1. Methodological framework for estimating the disease burden of atrial fibrillation and flutter. The flowchart illustrates two main analytical pathways: (1) overall disease burden estimation using standardized data, ICD mapping, and cause-of-death databases to calculate DALYs (orange boxes), and (2) smoking-attributable burden assessment through prevalence data extraction, exposure modeling, and population attributable fraction (PAF) calculations (blue boxes). EMR: excess mortality rate; CSMR: cause-specific mortality rate; YLD: years

lived with disability; YLL: years of life lost; ST-GPR: spatiotemporal Gaussian process regression.

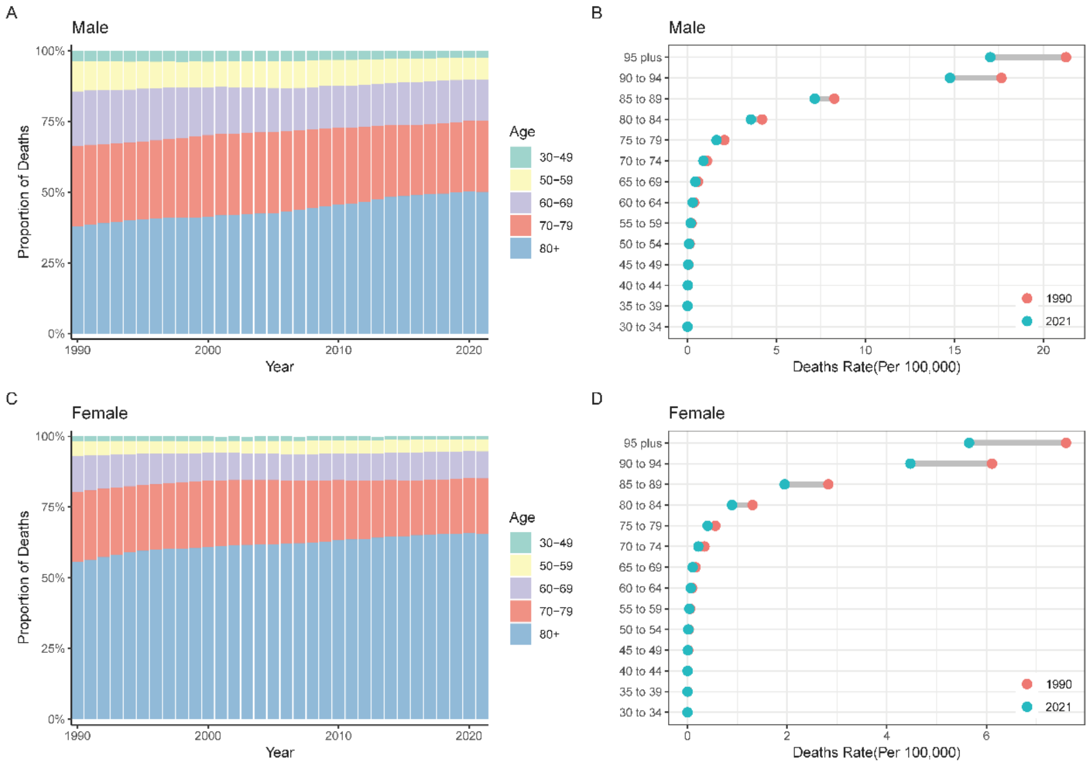

Figure S2. The temporal change in the sex specific age distribution and death rates of AF/AFL attributable to tobacco globally from 1990 to 2021. (A) Age specific proportion of AF/AFL deaths for males. (B) Comparison of AF/AFL death rates (per 100,000) for males from 1990 to 2021. (C) Age-specific proportion of AF/AFL deaths for females. (D) Comparison of AF/AFL death rates (per 100,000) for females from 1990 to 2021.

Abbreviation: AF/AFL, atrial fibrillation and atrial flutter.

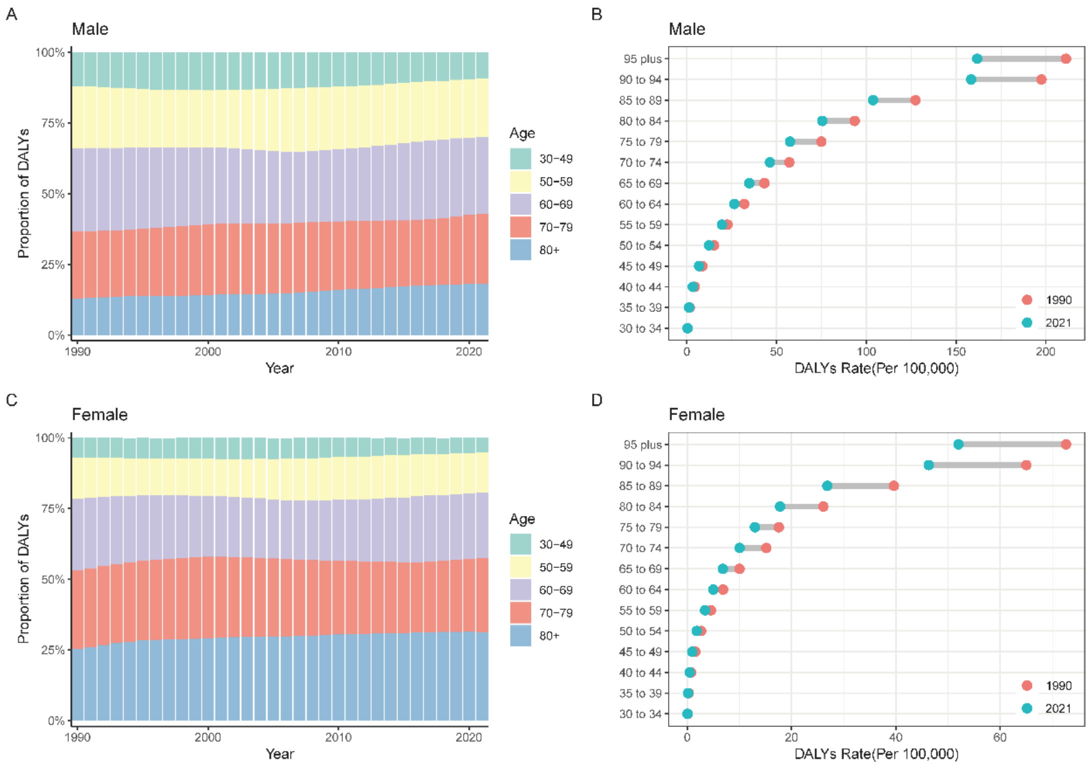

Figure S3. The temporal change in the sex specific age distribution and DALYs rates of AF/AFL attributable to tobacco globally from 1990 to 2021. (A) Age-specific proportion of AF/AFL DALYs for males. (B) Comparison of DALYs rates (per 100,000) for males in 1990 vs 2021. (C) Age specific proportion of AF/AFL DALYs for females. (D) Comparison of DALYs rates (per 100,000) for females in 1990 vs 2021.

Abbreviation: AF/AFL, atrial fibrillation and atrial flutter; DALYs, disability adjusted life years.

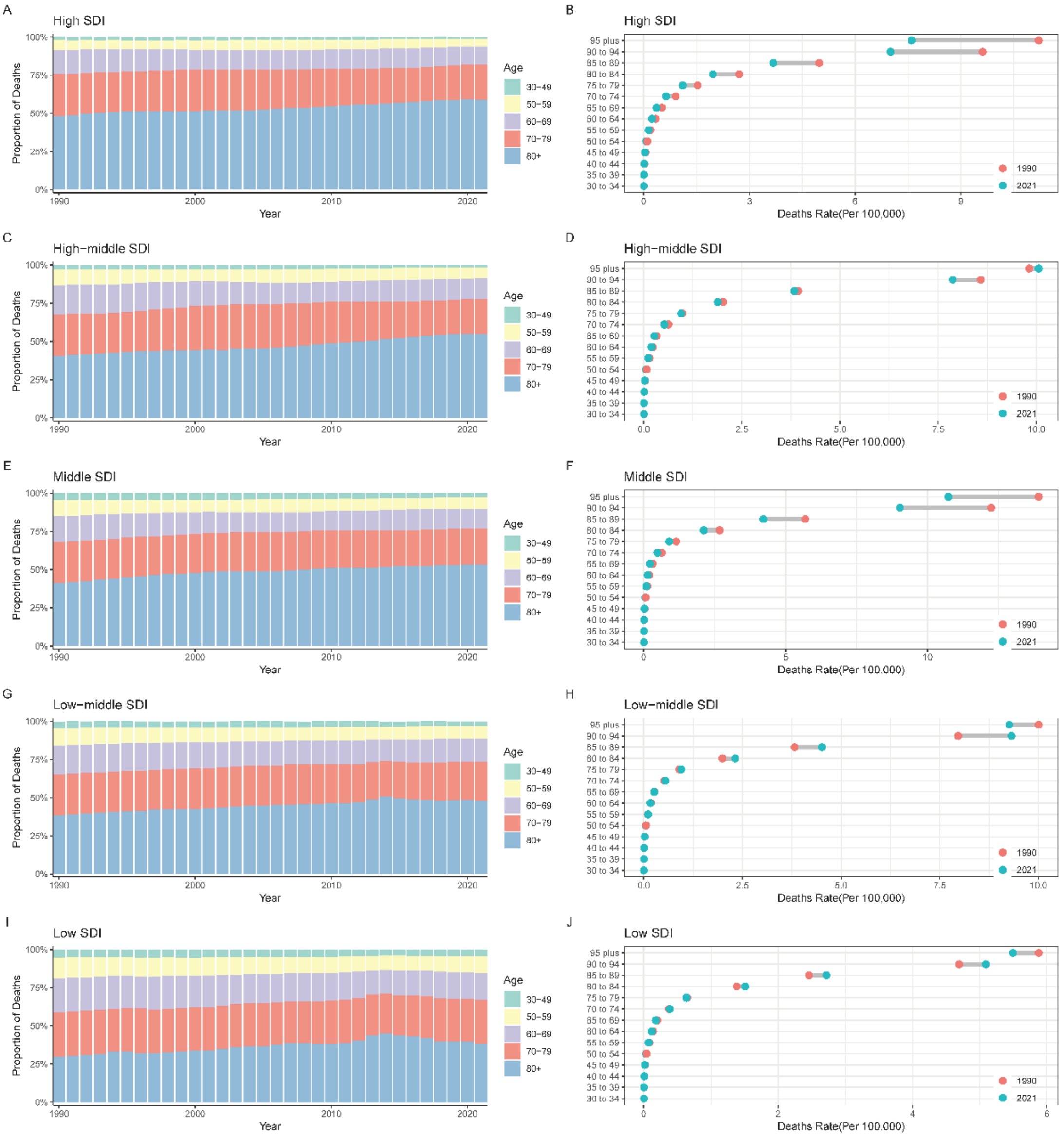

Figure S4. The temporal change in the age distribution and death rates of AF/AFL attributable to tobacco across five SDI regions globally from 1990 to 2021. (A, B) High SDI: Age-specific proportion of AF/AFL deaths (A) and comparison of AF/AFL death rates (per 100,000) in 1990 vs 2021 (B). Comparison of AF/AFL death rates (per 100,000) for different age groups in the high SDI region between 1990 and 2021. (C) Age-specific proportion of AF/AFL deaths attributable to tobacco in the high-middle SDI region from 1990 to 2021. (D). Comparison of AF/AFL death rates (per 100,000) for different age groups in the high-middle SDI region between 1990 and 2021. (E) Age specific proportion of AF/AFL deaths attributable to tobacco in the middle SDI region from 1990 to 2021. (F) Comparison of AF/AFL death rates (per 100,000) for different age groups in the middle SDI region between 1990 and 2021. (G) Age specific proportion of AF/AFL deaths attributable to tobacco in the low-middle SDI region from 1990 to 2021. (H) Comparison of AF/AFL death rates (per 100,000) for different age groups in

the low-middle SDI region between 1990 and 2021. (I) Age specific proportion of AF/AFL deaths attributable to tobacco in the low SDI region from 1990 to 2021. (J). Comparison of AF/AFL death rates (per 100,000) for different age groups in the low-SDI region between 1990 and 2021.

Abbreviation: AF/AFL, atrial fibrillation and atrial flutter; SDI, sociodemographic index.

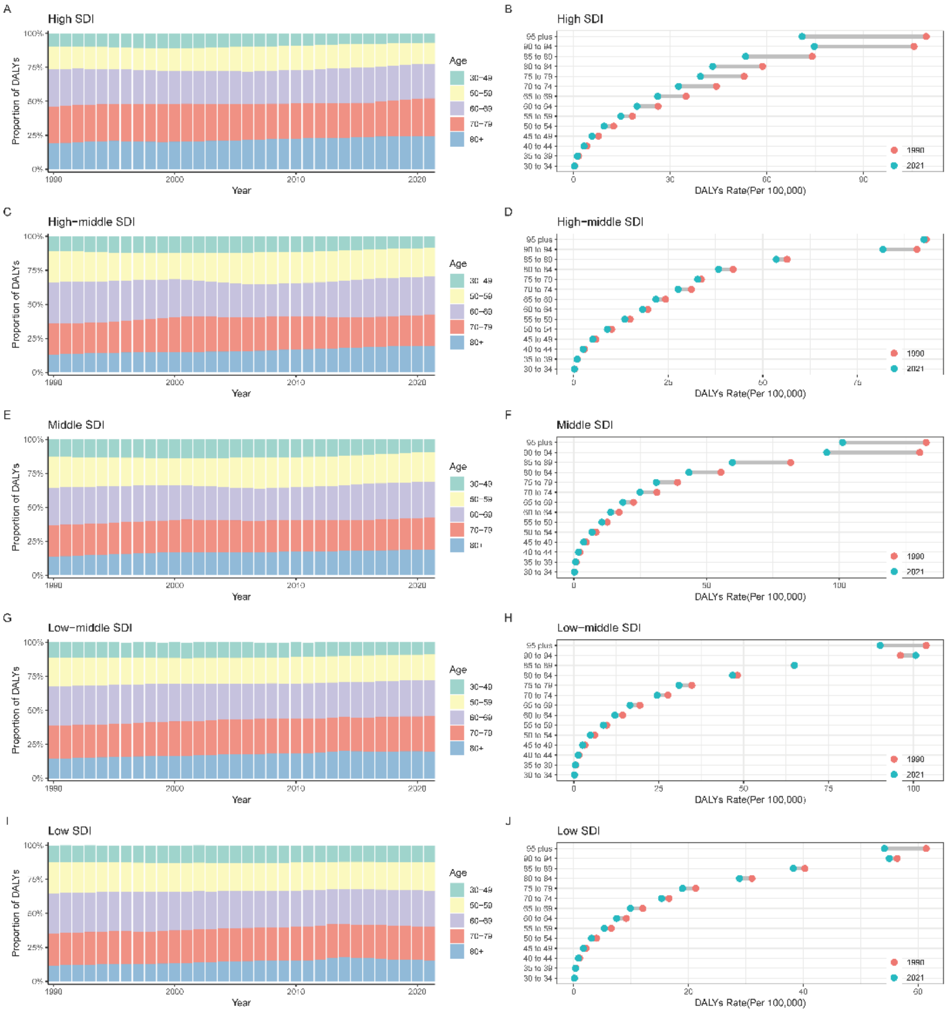

Figure S5. The temporal change in the age distribution and DALYs rates of AF/AFL attributable to tobacco across five SDI regions globally from 1990 to 2021. (A) Age specific proportion of AF/AFL DALYs in the high SDI region. (B) Comparison of AF/AFL DALYs rates (per 100,000) in the high SDI region between 1990 and 2021. (C) Agespecific proportion of AF/AFL DALYs in the high-middle SDI region. (D) Comparison of AF/AFL DALYs rates (per 100,000) in the high-middle SDI region between 1990 and 2021. (E) Age specific proportion of AF/AFL DALYs in the middle SDI region. (F) Comparison of AF/AFL DALYs rates (per 100,000) in the middle SDI region between 1990 and 2021. (G) Age specific proportion of AF/AFL DALYs in the low-middle SDI region. (H) Comparison of AF/AFL DALYs rates (per 100,000) in the low-middle SDI region between 1990 and 2021. (I) Age-specific proportion of AF/AFL DALYs in the low SDI region. (J) Comparison of AF/AFL DALYs rates (per 100,000) in the low SDI region between 1990 and 2021.

Abbreviation: AF/AFL, atrial fibrillation and atrial flutter; DALYs, disability adjusted life years; SDI, sociodemographic index.

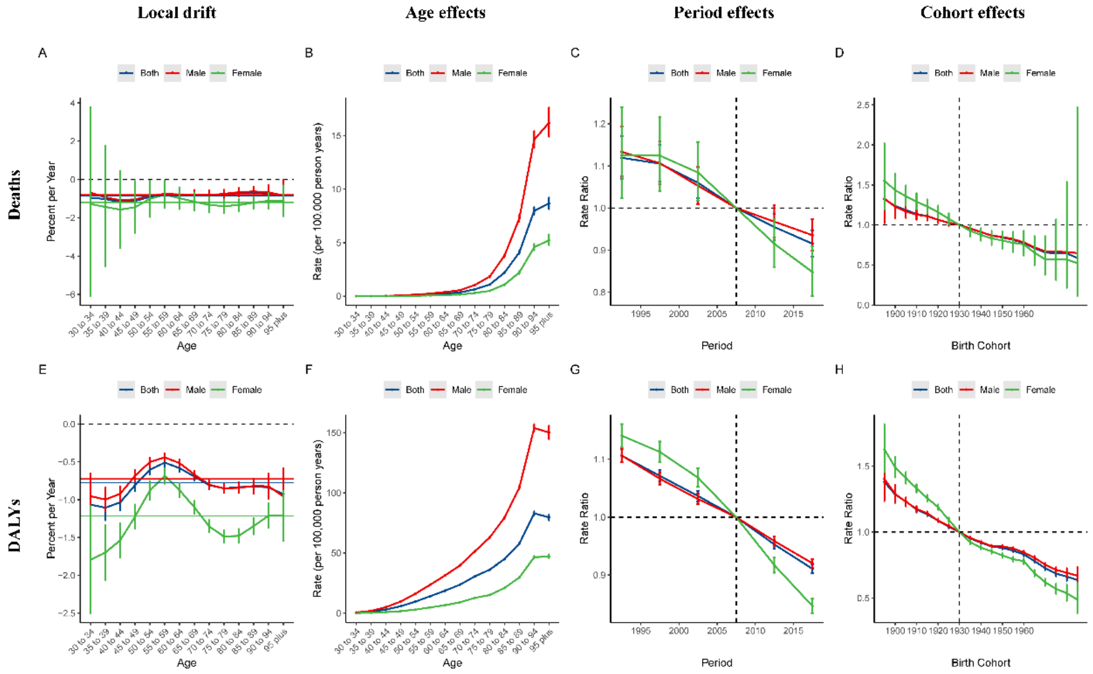

Figure S6. Age-Period-Cohort analysis of AF/AFL mortality and DALYs attributable to tobacco use globally, 1990–2021. (A) Local drift analysis of AF/AFL mortality. (B) Age effects on AF/AFL mortality. (C) Period effects on AF/AFL mortality. (D) Cohort effects on AF/AFL mortality. (E) Local drift analysis of AF/AFL DALYs. (F) Age effects on AF/AFL DALYs. (G) Period effects on AF/AFL DALYs. (H) Cohort effects on AF/AFL DALYs. The vertical error bars indicate the 95% CIs for the estimated rates and rate ratios.

Abbreviation: AF/AFL, atrial fibrillation and atrial flutter; DALYs, disability adjusted life years; SDI, sociodemographic index, Cis, confidence intervals.

A

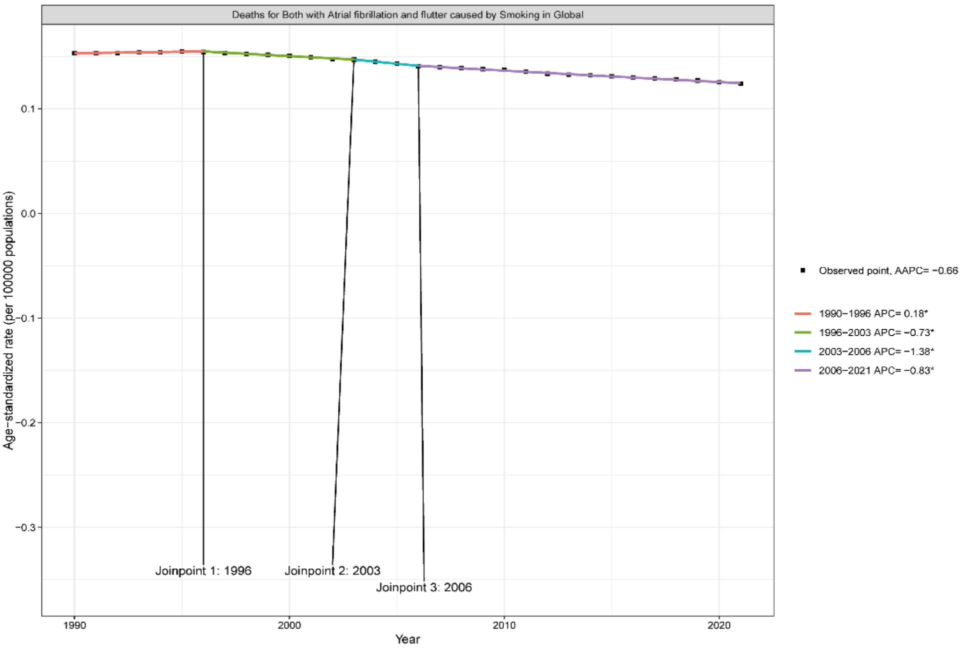

B

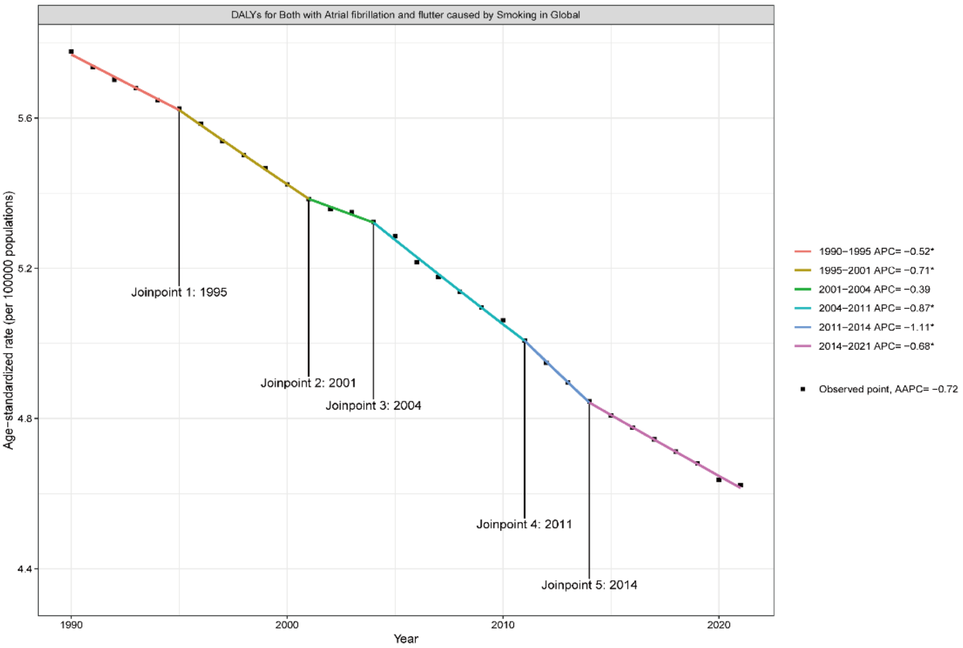

Figure S7. Joinpoint regression analysis of age-standardized rates of AF/AFL attributable to tobacco globally from 1990 to 2021. (A) ASMR of AF/AFL attributable to tobacco with joinpoint regression and estimated APC during different periods. (B) ASDR of AF/AFL attributable to tobacco with joinpoint regression and estimated APC during different periods.

Abbreviation: AF/AFL, atrial fibrillation and atrial flutter; ASMR, age standardized mortality rates; ASDR, age standardized DALYs rates; DALYs, disability-adjusted life years; APC, annual percentage change; AAPC, average annual percentage change.

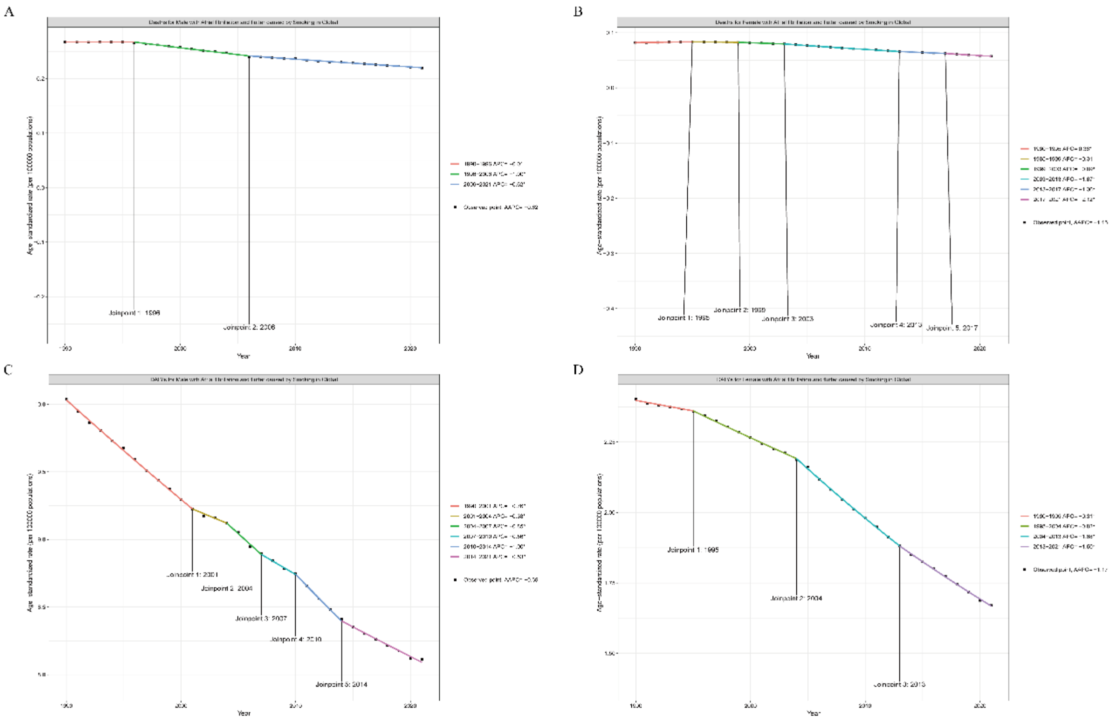

Figure S8. Joinpoint regression analysis of age-standardized death and DALYs rates of AF/AFL attributable to tobacco stratified by sex at the global level, 1990–2021. (A) ASMR of AF/AFL attributable to tobacco in males with joinpoint regression and APC in different periods. (B) ASMR of AF/AFL attributable to tobacco in females with joinpoint regression and APC in different periods. (C) ASDR of AF/AFL attributable to tobacco in males with joinpoint regression and APC in different periods. (D) ASDR of AF/AFL attributable to tobacco in females with joinpoint regression and APC in different periods.

Abbreviation: AF/AFL, atrial fibrillation and atrial flutter; ASMR, age standardized mortality rates; ASDR, age standardized DALYs rates; DALYs, disability adjusted life years; APC, annual percentage change; AAPC, average annual percentage change.

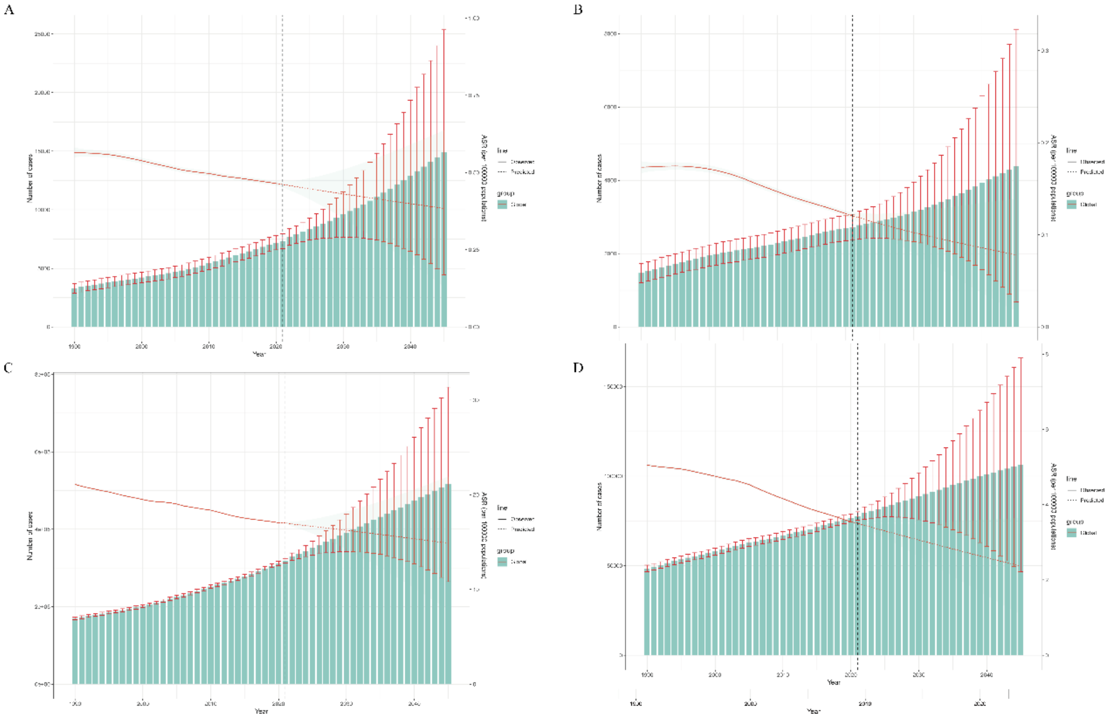

Figure S9. Observed and forecasted numbers and ASR of AF/AFL attributable to tobacco, stratified by sex at the global level, 1990–2049. (A) Observed and projected deaths (bars, left axis) and ASMR (lines, right axis) of AF/AFL attributable to tobacco in males, with 95% uncertainty intervals. (B) Observed and projected deaths (bars, left axis) and ASMR (lines, right axis) of AF/AFL attributable to tobacco in females, with 95% uncertainty intervals. (C) Observed and projected DALYs (bars, left axis) and ASDR (lines, right axis) of AF/AFL attributable to tobacco in males, with 95% uncertainty intervals. (D) Observed and projected DALYs (bars, left axis) and ASDR (lines, right axis) of AF/AFL attributable to tobacco in females, with 95% uncertainty intervals.

Abbreviation: AF/AFL, atrial fibrillation and atrial flutter; ASMR, age standardized mortality rates; DALYs, disability-adjusted life years; ASDR, age standardized DALYs rates; ASR, age-standardized rate.

Table S1. Deaths, ASMR, DALYs, ASDR, and EAPCs of AF/AFL attributable to tobacco in 204 countries and territories, 1990–2021.

characteristic	Deaths 1990	ASMR 1990	DALYs 1990	ASDR 1990	Deaths 2021	ASMR 2021	DALYs 2021	ASDR 2021	EAPC ASMR	EAPC ASDR
Afghanistan	1.4 (0.5-2.8)	0 (0-0.1)	56 (27.3-96.4)	0.9 (0.4-1.5)	3 (1.4-5.2)	0 (0-0.1)	133.4 (72.8-215.6)	1.4 (0.7-2.2)	1.71 (1.44-1.98)	1.61 (1.36-1.87)
Albania	3 (1.7-4.5)	0.2 (0.1-0.3)	126.8 (74.2-195.4)	6.6 (3.8-10.2)	9.4 (4.9-15.5)	0.2 (0.1-0.4)	332.3 (185.3-509.3)	7.7 (4.3-11.7)	1.09 (0.94-1.24)	0.81 (0.71-0.9)
Algeria	8.9 (4.5-14.2)	0.2 (0.1-0.3)	333.2 (187-518.2)	3.9 (2.2-6.2)	28.3 (15.3-45.9)	0.2 (0.1-0.3)	918.2 (514.2-1406)	3.4 (1.9-5.2)	-0.08 (-0.33-0.16)	-0.39 (-0.5--0.27)
American Samoa	0 (0-0.1)	0.2 (0.1-0.3)	1.8 (1.1-2.7)	7.4 (4.3-10.8)	0.1 (0-0.1)	0.2 (0.1-0.3)	3.8 (2.3-5.7)	7.6 (4.4-11.2)	0.06 (-0.01-0.14)	0.07 (0.04-0.1)
Andorra	0.1 (0-0.1)	0.2 (0.1-0.3)	4.6 (2.6-7)	8.1 (4.5-12.4)	0.2 (0.1-0.3)	0.1 (0.1-0.2)	8.4 (4.5-12.9)	5.4 (2.9-8.4)	-1.66 (-1.86--1.47)	-1.39 (-1.47--1.31)
Angola	2.5 (1.2-4.2)	0.1 (0-0.1)	108.5 (59.2-172.8)	2.9 (1.6-4.5)	7.1 (3.8-11.3)	0.1 (0-0.1)	308.2 (169.4-482.4)	2.6 (1.4-4)	-0.1 (-0.36-0.17)	-0.19 (-0.42-0.04)
Antigua and Barbuda	0.1 (0-0.1)	0.1 (0.1-0.2)	1.7 (1-2.6)	3.2 (1.8-5)	0.1 (0-0.1)	0.1 (0-0.1)	3.1 (1.7-4.9)	2.9 (1.7-4.6)	-0.41 (-0.66--0.16)	-0.36 (-0.5--0.23)
Argentina	24.1 (14.6-	0.1 (0-	1246.3 (734.8-	3.9 (2.3-	40.1 (23.3-	0.1 (0-	1610.7 (936-	2.9 (1.7-	0.32 (-0.1-	-0.56

	36.3)	0.1)	1887)	5.9)	62.5)	0.1)	2436.1)	4.5)	0.74)	(-0.76- -0.35)
Armenia	1.3 (0.8-2)	0.1 (0- 0.1)	111.4 (63- 174.8)	4 (2.3- 6.2)	5 (3-7.2)	0.1 (0.1- 0.2)	234.1 (138- 350.9)	5.4 (3.1- 8.1)	2.92 (2.38- 3.47)	1.23 (1.05- 1.42)
Australia	31 (18.5- 46.1)	0.2 (0.1- 0.2)	1477.7 (869.7- 2200.1)	7.7 (4.5- 11.4)	51.8 (28.3- 80.7)	0.1 (0.1- 0.2)	1893.6 (1051.7- 2978.3)	4.4 (2.4- 6.9)	-1.46 (-1.66- -1.25)	-1.62 (-1.71- -1.54)
Austria	16 (9.3- 23.4)	0.1 (0.1- 0.2)	628.3 (373- 939.9)	5.6 (3.3- 8.4)	35.2 (19.4- 55.4)	0.2 (0.1- 0.3)	1467.5 (863- 2253.8)	8.5 (5- 13.1)	0.92 (0.42- 1.41)	1.54 (1.25- 1.83)
Azerbaijan	2.2 (1.3- 3.4)	0 (0- 0.1)	166.5 (94.2- 255.5)	3.2 (1.8- 5.1)	5.4 (2.9-8.8)	0.1 (0- 0.1)	420.8 (228- 663.1)	4 (2.2- 6.4)	1.41 (1.12- 1.69)	0.88 (0.7- 1.05)
Bahamas	0.1 (0.1- 0.2)	0.1 (0- 0.1)	4.7 (2.7-7.2)	3.1 (1.7- 4.7)	0.3 (0.2-0.5)	0.1 (0- 0.1)	12.1 (6.7-18.8)	3 (1.6- 4.6)	0.22 (0.06- 0.38)	-0.01 (-0.07- 0.05)
Bahrain	0.1 (0.1- 0.2)	0.1 (0.1- 0.3)	5.1 (2.9-8.1)	3.6 (1.9- 6)	0.4 (0.2-0.7)	0.1 (0- 0.2)	22 (12.1-35.5)	2.8 (1.5- 4.6)	-1.13 (-1.46- -0.8)	-0.93 (-1.04- -0.82)
Bangladesh	64.8 (27.5- 104.4)	0.2 (0.1- 0.3)	2417.2 (1317- 3781.8)	5.8 (3.2- 9.2)	248.2 (131.7- 404.5)	0.3 (0.1- 0.4)	7511.3 (4183.6- 11403.3)	6.2 (3.5- 9.5)	0.8 (0.53- 1.07)	0.22 (0.12- 0.33)
Barbados	0.3 (0.1- 0.4)	0.1 (0- 0.1)	8.2 (4.5-12.8)	2.8 (1.5- 4.3)	0.4 (0.2-0.6)	0.1 (0- 0.1)	11.8 (6.8-18.7)	2.3 (1.3- 3.7)	-0.76 (-1.07-	-0.87 (-1.08-

									-0.45)	-0.65)
Belarus	11.6 (5.9-19.3)	0.1 (0-0.2)	644.2 (360.9-997.4)	5 (2.8-7.8)	18.8 (10.7-27.4)	0.1 (0.1-0.2)	922.3 (530.8-1391.5)	5.8 (3.3-8.9)	0.54 (0.47-0.61)	0.48 (0.42-0.54)
Belgium	26.1 (15.2-38.6)	0.2 (0.1-0.2)	1063.8 (596.8-1641.5)	7.1 (4-10.9)	30.5 (16.5-46.7)	0.1 (0.1-0.2)	1085.2 (629.8-1692.4)	5 (2.9-7.9)	-1.23 (-1.58--0.88)	-0.98 (-1.13--0.82)
Belize	0.1 (0-0.2)	0.1 (0.1-0.2)	3.9 (2.2-6)	4.3 (2.4-6.6)	0.3 (0.1-0.4)	0.1 (0.1-0.2)	11.6 (6.6-18)	3.9 (2.2-6.1)	-0.16 (-0.48-0.16)	-0.2 (-0.31--0.08)
Benin	0.6 (0.3-0.9)	0 (0-0.1)	26.3 (14.3-40.4)	1.4 (0.7-2.1)	0.9 (0.4-1.4)	0 (0-0)	43 (23-70.1)	0.9 (0.5-1.4)	-1.76 (-1.97--1.56)	-1.74 (-1.95--1.53)
Bermuda	0.1 (0-0.1)	0.1 (0.1-0.2)	2.5 (1.4-3.8)	4.1 (2.3-6.3)	0.1 (0.1-0.2)	0.1 (0-0.1)	4.6 (2.6-7.2)	3.4 (1.9-5.4)	-1.27 (-1.34--1.21)	-0.61 (-0.65--0.56)
Bhutan	0.1 (0-0.1)	0.1 (0-0.1)	3.8 (1.9-6.2)	1.9 (1-3.1)	0.4 (0.2-0.7)	0.1 (0-0.1)	11.9 (6.3-19)	2.1 (1.1-3.4)	1.42 (1.34-1.49)	0.4 (0.36-0.45)
Bolivia (Plurinational State of)	2.8 (1.4-4.9)	0.1 (0.1-0.2)	120.3 (66.2-189.7)	3.9 (2.1-6.1)	7.1 (3.8-12)	0.1 (0-0.2)	305 (166-467.5)	3.4 (1.9-5.2)	-0.12 (-0.33-0.09)	-0.07 (-0.29-0.15)
Bosnia and Herzegovina	4.8 (2.7-7.2)	0.1 (0.1-0.2)	230.6 (132.7-352.7)	5.7 (3.2-8.9)	11.9 (6.2-18.8)	0.2 (0.1-0.3)	417.6 (232.6-632.3)	6.8 (3.7-10.1)	1.07 (0.73-1.41)	0.75 (0.48-1.03)

Botswana	0.5 (0.3-0.7)	0.1 (0.1-0.2)	22.8 (12.8-35.1)	4.4 (2.4-6.8)	1 (0.5-1.6)	0.1 (0-0.2)	51.4 (29.1-78.7)	3.6 (2-5.5)	-0.87 (-0.99--0.74)	-0.68 (-0.76--0.61)
Brazil	142.4 (86.5-207.2)	0.2 (0.1-0.3)	7576.4 (4379.7-11451.3)	8.9 (5.2-13.3)	285.7 (167.1-430.6)	0.1 (0.1-0.2)	12118.3 (6857.9-18679.5)	4.8 (2.7-7.5)	-2.36 (-2.51--2.21)	-2.14 (-2.22--2.06)
Brunei Darussalam	0.3 (0.1-0.4)	0.3 (0.2-0.5)	9.6 (5.5-14.5)	9.3 (5.3-13.8)	0.4 (0.2-0.6)	0.2 (0.1-0.3)	17.7 (10.1-26.5)	4.9 (2.7-7.4)	-1.62 (-1.94--1.31)	-1.87 (-2.14--1.61)
Bulgaria	15.7 (8.8-23.4)	0.1 (0.1-0.2)	788.6 (449-1181.3)	6.3 (3.6-9.5)	22.9 (12.8-34)	0.2 (0.1-0.2)	857.3 (493.9-1250.7)	6.6 (3.8-9.5)	0.38 (0.24-0.51)	0.09 (0.02-0.17)
Burkina Faso	1 (0.4-1.7)	0 (0-0.1)	47 (24.8-75.3)	1.1 (0.6-1.8)	2.5 (1.1-4.2)	0 (0-0.1)	100.3 (52.3-163.5)	1.1 (0.6-1.8)	0.5 (0.36-0.65)	-0.01 (-0.11-0.09)
Burundi	1.6 (0.7-3)	0.1 (0-0.2)	65.8 (33.9-112.3)	2.9 (1.5-5)	1.6 (0.6-2.9)	0 (0-0.1)	82.9 (41.5-139.3)	1.7 (0.9-2.8)	-2.43 (-3.06--1.8)	-1.78 (-2.29--1.28)
Cabo Verde	0.1 (0-0.1)	0 (0-0.1)	3.2 (1.7-5)	1.4 (0.8-2.2)	0.1 (0.1-0.2)	0 (0-0.1)	4.8 (2.6-7.7)	1.1 (0.6-1.7)	-1.14 (-1.59--0.68)	-1.17 (-1.42--0.91)
Cambodia	4.9 (2.7-8.1)	0.2 (0.1-0.3)	255.5 (152.9-385.2)	6.5 (3.9-9.7)	16.3 (8.9-26.1)	0.2 (0.1-0.4)	698.1 (399.3-1063.8)	6.5 (3.7-10.1)	0.41 (0.31-0.51)	-0.28 (-0.42--0.14)
Cameroon	1.6 (0.8-	0.1 (0-	66.6 (36.4-	1.6 (0.9-	3.5 (1.8-5.9)	0 (0-	156.1 (85.6-	1.3 (0.7-	-0.74	-0.7

	2.5)	0.1)	103.5)	2.5)		0.1)	243.8)	2.1)	(-0.87--0.62)	(-0.87--0.54)
Canada	67.6 (39.7-100.1)	0.2 (0.1-0.3)	3236 (1843-5033.2)	10 (5.7-15.4)	96.4 (53.2-149.5)	0.1 (0.1-0.2)	3864.8 (2136.8-6162.9)	5.4 (3-8.5)	-2.2 (-2.32--2.07)	-2.04 (-2.16--1.91)
Central African Republic	0.6 (0.3-1.1)	0.1 (0-0.1)	25.9 (12.6-43.6)	2.3 (1.1-3.8)	0.9 (0.4-1.5)	0.1 (0-0.1)	40 (20.5-67)	1.7 (0.9-2.8)	-1.01 (-1.22--0.8)	-0.99 (-1.21--0.78)
Chad	1 (0.5-1.7)	0.1 (0-0.1)	42.3 (22-68.4)	1.6 (0.8-2.6)	1.9 (0.9-3.3)	0.1 (0-0.1)	76.7 (41.6-126.4)	1.5 (0.8-2.5)	-0.11 (-0.29-0.07)	-0.47 (-0.61--0.33)
Chile	6.5 (4-9.4)	0.1 (0-0.1)	384.4 (223.8-571.4)	3.7 (2.2-5.5)	14.8 (8.7-21.9)	0.1 (0-0.1)	741.4 (429.4-1131.9)	3 (1.7-4.6)	0.24 (-0.32-0.8)	-0.5 (-0.77--0.23)
China	935.7 (521.5-1434.4)	0.2 (0.1-0.3)	44181.9 (26152-65797.3)	6.2 (3.7-9.2)	2681.7 (1472.3-4093.2)	0.2 (0.1-0.2)	113500.6 (63199.6-171028.2)	5.5 (3.1-8.3)	-1.12 (-1.21--1.04)	-0.29 (-0.38--0.2)
Colombia	13.7 (8.5-20.3)	0.1 (0.1-0.1)	708.9 (405.6-1098.1)	4 (2.3-6.2)	31.6 (17.1-51.2)	0.1 (0-0.1)	1346.5 (765.1-2128.7)	2.4 (1.4-3.8)	-2.19 (-2.36--2.02)	-1.88 (-2.01--1.75)
Comoros	0.2 (0.1-0.3)	0.1 (0-0.2)	6.2 (3.2-10.3)	3.6 (1.8-5.9)	0.3 (0.1-0.7)	0.1 (0-0.2)	11.6 (5.6-19.7)	2.6 (1.3-4.5)	-1.15 (-1.32--0.98)	-1.27 (-1.41--1.13)
Congo	0.7 (0.3-1)	0.1 (0-0.1)	24.3 (12.9-38)	2.4 (1.3-3.7)	1.7 (0.9-2.7)	0.1 (0-0.1)	70.9 (39.8-107.8)	2.7 (1.5-4.2)	0.57 (0.32-	0.65 (0.42-

									0.83)	0.87)
Cook Islands	0 (0-0)	0.2 (0.1-0.3)	1 (0.6-1.5)	7.8 (4.5-11.5)	0 (0-0.1)	0.2 (0.1-0.3)	1.6 (0.9-2.4)	6.3 (3.5-9.8)	-1.16 (-1.24--1.08)	-0.79 (-0.85--0.74)
Costa Rica	2.2 (1.2-3.2)	0.1 (0.1-0.2)	90.3 (50.8-139.5)	5.3 (3-8.3)	5.1 (2.9-7.8)	0.1 (0.1-0.1)	192.4 (105.8-302.2)	3.5 (1.9-5.5)	-1.59 (-1.88--1.3)	-1.5 (-1.68--1.32)
Coted'Ivoire	1.3 (0.6-2.1)	0.1 (0-0.1)	59.6 (32.7-94)	1.7 (0.9-2.7)	3.6 (1.9-5.7)	0.1 (0-0.1)	161.7 (85.1-250)	1.6 (0.8-2.5)	-0.97 (-1.26--0.68)	-0.77 (-1.03--0.51)
Croatia	7.5 (4.3-11.1)	0.1 (0.1-0.2)	290.3 (171.1-434.4)	4.9 (2.9-7.3)	15 (8.4-23.2)	0.1 (0.1-0.2)	424.3 (246.2-644)	4.8 (2.8-7.2)	0.61 (0.26-0.96)	0.51 (0.26-0.77)
Cuba	17.6 (9.9-27.5)	0.2 (0.1-0.3)	739.3 (424.3-1116.4)	7.4 (4.2-11.1)	28 (15.3-42.9)	0.1 (0.1-0.2)	1072.1 (602-1638.2)	5.5 (3.1-8.3)	-1.36 (-1.45--1.28)	-1.17 (-1.27--1.07)
Cyprus	2 (1.1-3.2)	0.4 (0.2-0.6)	69.9 (40.2-105.3)	9.7 (5.6-14.7)	3.4 (1.9-5.2)	0.2 (0.1-0.3)	111.4 (62.8-168.1)	5.5 (3.1-8.3)	-2.75 (-2.87--2.63)	-1.81 (-1.9--1.71)
Czechia	20.9 (12.3-31.3)	0.2 (0.1-0.2)	949.2 (546.3-1455.3)	6.9 (4-10.6)	33 (18.7-48.8)	0.1 (0.1-0.2)	1536.2 (881.5-2291.2)	7.3 (4.2-10.8)	-0.12 (-0.26-0.02)	0.32 (0.07-0.57)
Democratic People's Republic of Korea	14.1 (7.8-21.8)	0.1 (0.1-0.2)	757.7 (433.8-1134.8)	4.8 (2.8-7.2)	28.8 (15.1-43.8)	0.1 (0.1-0.2)	1436.2 (802.1-2204.7)	4.3 (2.4-6.5)	-0.74 (-0.83--0.66)	-0.42 (-0.45--0.4)

Democratic Republic of the Congo	6 (2.6-10.3)	0.1 (0-0.1)	279.1 (145.7-448.1)	1.8 (0.9-2.9)	12.5 (5.8-21.2)	0 (0-0.1)	582.7 (309.1-933.3)	1.5 (0.8-2.4)	-0.73 (-1.18--0.29)	-0.57 (-0.89--0.25)
Denmark	33.5 (19.4-48.4)	0.4 (0.2-0.6)	1104.6 (636-1683.1)	13.6 (7.9-20.6)	35.5 (19.8-53.4)	0.3 (0.1-0.4)	1092.1 (624.8-1643.8)	9.3 (5.4-14)	-1.54 (-2.04--1.04)	-1.71 (-2.04--1.39)
Djibouti	0.1 (0.1-0.2)	0.2 (0.1-0.3)	5.5 (3.2-8.5)	4.5 (2.6-6.9)	0.6 (0.3-0.9)	0.2 (0.1-0.3)	25.4 (13.6-39.9)	4.4 (2.4-6.9)	-0.03 (-0.15-0.1)	0.07 (-0.02-0.15)
Dominica	0.1 (0-0.1)	0.1 (0.1-0.2)	2 (1.1-3.1)	3.4 (1.9-5.3)	0.1 (0-0.1)	0.1 (0.1-0.2)	2.8 (1.5-4.4)	3.3 (1.8-5.2)	-0.44 (-0.5--0.38)	-0.11 (-0.21--0.02)
Dominican Republic	6.7 (3.7-10.3)	0.3 (0.1-0.4)	222.9 (125-341.8)	7 (3.9-10.8)	18.8 (9.3-31.7)	0.2 (0.1-0.3)	553.8 (304.6-884.4)	5.7 (3.1-9.1)	-0.84 (-1.34--0.33)	-0.63 (-0.93--0.32)
Ecuador	5.9 (3.4-8.8)	0.1 (0.1-0.2)	237.1 (136.6-367.9)	4.7 (2.7-7.3)	9.6 (5.4-14.7)	0.1 (0-0.1)	431.7 (241-663)	2.7 (1.5-4.1)	-2.43 (-2.54--2.33)	-1.75 (-1.81--1.68)
Egypt	16.7 (9.1-26.2)	0.1 (0.1-0.2)	705.7 (396.5-1070.9)	3.3 (1.9-5)	41.6 (23.5-61.5)	0.1 (0.1-0.2)	1919.6 (1101.8-2903.9)	3.8 (2.2-6)	0.47 (0.3-0.65)	0.65 (0.58-0.72)
El Salvador	1.4 (0.8-2.1)	0 (0-0.1)	60.8 (33.7-94.7)	2 (1.1-3.2)	3.5 (1.9-5.7)	0.1 (0-0.1)	139.2 (79.5-213.6)	2.3 (1.3-3.5)	0.34 (0.23-0.45)	0.46 (0.39-0.53)
Equatorial Guinea	0.1 (0.1-	0.1 (0-	4.6 (2.4-7.4)	2.4 (1.2-	0.3 (0.1-0.5)	0.1 (0-	11.9 (6.2-19.2)	2.3 (1.2-	-0.09	-0.11

	0.2)	0.1)		3.8)		0.1)		3.7)	(-0.33-0.15)	(-0.31-0.09)
Eritrea	0.3 (0.2-0.6)	0 (0-0.1)	17.8 (9.3-30.3)	1.3 (0.7-2.2)	0.7 (0.3-1.1)	0 (0-0.1)	32.6 (16.7-52.7)	1 (0.5-1.7)	-0.65 (-0.87--0.42)	-1.01 (-1.22--0.81)
Estonia	1.7 (1-2.6)	0.1 (0-0.1)	96.6 (55.1-146.7)	4.7 (2.7-7.2)	3.4 (2-5.2)	0.1 (0.1-0.2)	132 (75.8-200.4)	5.4 (3.1-8.2)	0.78 (0.57-1)	0.51 (0.31-0.72)
Eswatini	0.2 (0.1-0.3)	0.1 (0-0.2)	7.2 (4-11.1)	2.8 (1.5-4.4)	0.3 (0.1-0.4)	0.1 (0-0.1)	10.6 (5.8-16.9)	2.1 (1.1-3.3)	-0.69 (-1.01--0.37)	-0.89 (-1.09--0.69)
Ethiopia	4.4 (1.6-7.4)	0 (0-0)	228.7 (113.8-370.6)	1.1 (0.6-1.8)	7.9 (3.4-14.8)	0 (0-0)	417 (212.9-671.2)	1 (0.5-1.6)	-0.12 (-0.49-0.26)	-0.13 (-0.44-0.18)
Fiji	0.6 (0.4-0.8)	0.2 (0.1-0.3)	29.6 (17.3-43.1)	7.3 (4.3-10.8)	1.1 (0.6-1.7)	0.2 (0.1-0.2)	54.2 (30.2-81)	6.4 (3.7-9.6)	-0.52 (-0.64--0.4)	-0.46 (-0.53--0.39)
Finland	11.6 (6.6-17.4)	0.2 (0.1-0.2)	596.5 (333.7-923.5)	8.7 (4.9-13.5)	13.2 (7.4-19.9)	0.1 (0.1-0.1)	567.1 (320.9-893.7)	5.1 (3-8)	-1.75 (-2.09--1.41)	-1.67 (-1.85--1.5)
France	148.7 (86.3-230.3)	0.2 (0.1-0.3)	5754.7 (3180.4-8843.8)	7.1 (4-10.8)	190.9 (104.4-298.7)	0.1 (0.1-0.2)	6825.2 (3879.4-10613.7)	5.4 (3-8.2)	-1.37 (-1.48--1.27)	-0.9 (-0.97--0.84)
Gabon	0.3 (0.2-0.5)	0.1 (0-0.1)	11.3 (6.1-17.5)	2 (1.1-3.2)	0.6 (0.3-0.9)	0.1 (0-0.1)	23.2 (12.7-35.3)	2.2 (1.2-3.4)	-0.12 (-0.21-	0.19 (0.14-

									-0.04)	0.24)
Gambia	0.2 (0.1-0.3)	0.1 (0-0.1)	7.9 (4.3-12.4)	2.4 (1.3-3.8)	0.4 (0.2-0.6)	0.1 (0-0.1)	15.5 (8.6-23.9)	1.7 (0.9-2.6)	-0.94 (-1.05--0.83)	-1.42 (-1.52--1.32)
Georgia	4.9 (2.8-7.6)	0.1 (0-0.1)	275.6 (160.7-416.6)	4.3 (2.5-6.6)	14.2 (8.4-20.8)	0.2 (0.1-0.3)	450.6 (271-654.4)	7.7 (4.7-11)	3.89 (3.15-4.63)	2.22 (1.88-2.55)
Germany	275.5 (154.1-409.1)	0.2 (0.1-0.3)	11061.3 (6319.5-16860.5)	8.9 (5.1-13.5)	468.6 (263.8-715.9)	0.2 (0.1-0.3)	15929.6 (9137.6-24424.5)	8.9 (5.2-13.6)	0.47 (0.2-0.73)	0.18 (-0.07-0.43)
Ghana	2.1 (1.1-3.3)	0.1 (0-0.1)	77 (44.1-120.3)	1.6 (0.9-2.5)	5.4 (2.8-9)	0.1 (0-0.1)	208.9 (118-333)	1.5 (0.8-2.5)	-0.24 (-0.4--0.08)	0.08 (-0.07-0.24)
Greece	27.1 (16-39.5)	0.2 (0.1-0.3)	1167 (678.4-1775.9)	7.9 (4.6-11.9)	54 (30.4-81.4)	0.2 (0.1-0.3)	1651.3 (929.8-2551.9)	7.2 (4.1-11)	-0.42 (-0.56--0.28)	-0.37 (-0.58--0.16)
Greenland	0.1 (0.1-0.2)	0.5 (0.3-0.8)	6.3 (3.8-9.1)	18.2 (11-26.4)	0.2 (0.1-0.3)	0.3 (0.2-0.5)	9.4 (5.3-14)	13.1 (7.4-19.5)	-1.07 (-1.16--0.97)	-0.9 (-0.94--0.85)
Grenada	0.1 (0-0.1)	0.1 (0-0.1)	2.2 (1.2-3.4)	3.1 (1.7-4.8)	0.1 (0-0.1)	0.1 (0-0.1)	3.6 (1.9-5.3)	3 (1.7-4.6)	-0.49 (-0.98-0)	-0.41 (-0.63--0.19)
Guam	0.1 (0.1-0.1)	0.1 (0.1-0.2)	4.7 (2.8-6.9)	5.4 (3.2-8)	0.2 (0.1-0.2)	0.1 (0-0.1)	10.2 (5.8-15.5)	5 (2.8-7.5)	-0.89 (-1.25--0.52)	-0.02 (-0.15-0.12)

Guatemala	2 (1.1-3)	0.1 (0.1-0.2)	100.7 (55.9-154.8)	3.2 (1.8-5.1)	6.2 (3.5-9.6)	0.1 (0-0.1)	267.3 (149.1-412)	2.5 (1.4-3.8)	-1.55 (-1.74--1.35)	-0.94 (-1.04--0.84)
Guinea	1.3 (0.5-2.2)	0.1 (0-0.1)	49.5 (27.2-78.9)	1.6 (0.9-2.6)	2.2 (1.1-3.5)	0.1 (0-0.1)	81.1 (42.7-130)	1.6 (0.8-2.5)	-0.05 (-0.13-0.03)	-0.18 (-0.24--0.12)
Guinea-Bissau	0.1 (0-0.1)	0 (0-0)	3.1 (1.6-5.2)	0.8 (0.4-1.4)	0.1 (0.1-0.2)	0 (0-0.1)	7 (3.7-11.1)	1 (0.5-1.6)	0.87 (0.64-1.11)	0.89 (0.63-1.15)
Guyana	0.3 (0.2-0.4)	0.1 (0-0.1)	13 (7.3-20.1)	3.4 (1.9-5.2)	0.5 (0.3-0.8)	0.1 (0-0.1)	22.8 (12.8-34.9)	3.5 (2-5.4)	0.55 (0.3-0.79)	0.21 (0.13-0.29)
Haiti	2.1 (1-3.6)	0.1 (0-0.1)	103.9 (54.3-167.8)	3.2 (1.6-5.1)	3.4 (1.6-6.1)	0.1 (0-0.1)	163.5 (87.2-270.8)	2.3 (1.2-3.7)	-0.68 (-0.76--0.6)	-1.09 (-1.2--0.98)
Honduras	1.7 (0.9-2.8)	0.1 (0.1-0.2)	85.1 (48.3-132.2)	4.3 (2.4-6.7)	7.3 (3.9-11.3)	0.2 (0.1-0.2)	281.7 (159.7-432.3)	4.7 (2.6-7.1)	1.29 (1.13-1.45)	0.33 (0.25-0.4)
Hungary	16.3 (9.8-23.3)	0.1 (0.1-0.2)	867.7 (503.3-1331.6)	6 (3.5-9.1)	17.1 (9.8-26.2)	0.1 (0-0.1)	795.4 (439.9-1187.2)	4.4 (2.5-6.5)	-0.75 (-1.03--0.46)	-0.86 (-1.02--0.7)
Iceland	0.7 (0.4-1.2)	0.2 (0.1-0.4)	22.7 (13.1-35.9)	7.9 (4.6-12.5)	1 (0.6-1.7)	0.2 (0.1-0.2)	31.2 (17.5-48.3)	5.4 (3-8.2)	-1.36 (-1.49--1.23)	-1.48 (-1.56--1.4)
India	285.1	0.1 (0-	16021 (8644.1-	3.9 (2.1-	942.8 (530.3-	0.1	34761 (19795.3-	3.2 (1.9-	0.66	-0.57

	(136.3-468.7)	0.2)	24697.1)	6.1)	1429.8)	(0.1-0.2)	53021.2)	4.9)	(0.43-0.88)	(-0.65--0.5)
Indonesia	88.7 (50.5-137.1)	0.1 (0.1-0.2)	5285.8 (3038.9-7913.4)	5.9 (3.4-9)	315.4 (179.5-486.4)	0.2 (0.1-0.4)	15629.4 (8886.8-23307.9)	7.4 (4.3-11)	1.69 (1.35-2.03)	0.71 (0.51-0.91)
Iran (Islamic Republic of)	7.9 (4.3-11.7)	0 (0-0.1)	444 (253.9-692.2)	1.8 (1-2.8)	31.7 (17-49.7)	0 (0-0.1)	1487.3 (822.5-2315.4)	1.9 (1.1-3)	0.57 (0.47-0.67)	0.47 (0.39-0.55)
Iraq	11.8 (6.6-18.4)	0.2 (0.1-0.3)	388.8 (222.4-588.7)	5 (2.9-7.6)	27 (14.9-43.9)	0.2 (0.1-0.3)	968.7 (547.8-1464.2)	4.5 (2.5-6.9)	-0.41 (-0.61--0.21)	-0.67 (-0.79--0.56)
Ireland	14.6 (8.7-21.4)	0.4 (0.2-0.6)	460.1 (268.6-697.7)	11.4 (6.7-17.1)	13.4 (7.1-20.6)	0.2 (0.1-0.2)	403.3 (225.8-618.3)	5.1 (2.8-7.8)	-2.96 (-3.22--2.69)	-2.92 (-3.09--2.76)
Israel	9.3 (5.4-14.1)	0.2 (0.1-0.3)	382.8 (216-608.6)	8.1 (4.6-12.6)	15.1 (8.2-23.3)	0.1 (0.1-0.2)	767 (429.6-1170.8)	6.4 (3.6-9.9)	-1.95 (-2.08--1.83)	-0.47 (-0.67--0.27)
Italy	118.7 (68.6-175.5)	0.1 (0.1-0.2)	6327.1 (3565.5-9766)	7.4 (4.2-11.3)	180.7 (99.9-280.6)	0.1 (0.1-0.1)	6702.1 (3706.8-10608.3)	4.8 (2.7-7.8)	-0.81 (-0.97--0.65)	-1.26 (-1.32--1.2)
Jamaica	2 (1.1-3)	0.1 (0.1-0.2)	76.3 (42.7-117.9)	4.4 (2.4-6.7)	3.3 (1.8-5.4)	0.1 (0.1-0.2)	113.6 (64.7-177.6)	3.6 (2.1-5.6)	-0.62 (-0.88--0.36)	-0.86 (-1.02--0.71)
Japan	255.3 (158.1-	0.2 (0.1-	11389.4 (6777.3-	6.7 (4-10)	340.6 (194.7-513)	0.1 (0-0.1)	10597.9 (6053.4-16483.9)	3.5 (2-5.2)	-3.47 (-3.91-	-2.66 (-2.91-

	367.3)	0.2)	16963.7)						-3.04)	-2.42)
Jordan	1.2 (0.7-1.8)	0.1 (0.1-0.2)	48.8 (29.1-75.5)	4.2 (2.5-6.5)	4.9 (2.8-7.5)	0.1 (0.1-0.2)	256.6 (145.4-389.4)	3.8 (2.2-5.8)	-0.86 (-1.01--0.71)	-0.37 (-0.43--0.3)
Kazakhstan	5.3 (3-7.8)	0 (0-0.1)	441.1 (240.3-663.5)	3.3 (1.8-5)	8.3 (4.9-11.9)	0.1 (0-0.1)	661.1 (356.3-1012)	3.5 (1.9-5.4)	0.16 (-0.11-0.43)	0.03 (-0.15-0.2)
Kenya	4.2 (2.1-7.1)	0.1 (0-0.1)	201.4 (106.7-314.7)	2.7 (1.4-4.2)	8.8 (4.2-15.3)	0.1 (0-0.1)	430.3 (234.8-694.8)	2 (1.1-3.2)	-1.39 (-1.56--1.21)	-1.38 (-1.51--1.24)
Kiribati	0.1 (0-0.1)	0.2 (0.1-0.3)	3.3 (2.1-4.8)	8.7 (5.2-12.8)	0.1 (0.1-0.2)	0.2 (0.1-0.3)	7.2 (4.3-10.6)	9.3 (5.5-14)	0.2 (-0.07-0.47)	0.08 (-0.14-0.3)
Kuwait	0.3 (0.2-0.4)	0.1 (0-0.1)	17.3 (9.8-26.9)	2.8 (1.6-4.3)	1.9 (1-2.9)	0.1 (0-0.1)	77.6 (43.6-120.5)	2.7 (1.5-4.1)	0.92 (0.41-1.42)	-0.06 (-0.31-0.19)
Kyrgyzstan	1.2 (0.7-1.8)	0 (0-0.1)	86.9 (49.8-133.7)	2.9 (1.7-4.5)	3.3 (2-4.7)	0.1 (0-0.1)	203.8 (117.9-310.2)	4.1 (2.4-6.3)	2.59 (2.17-3.02)	1.57 (1.39-1.75)
Lao People's Democratic Republic	2.2 (1.1-4)	0.2 (0.1-0.3)	116.7 (65.4-185.6)	6.3 (3.6-10.2)	6.7 (3.8-10.4)	0.2 (0.1-0.4)	289.3 (166.2-436.2)	7.1 (4.1-10.8)	0.97 (0.93-1.01)	0.42 (0.38-0.46)
Latvia	3.1 (1.8-4.5)	0.1 (0.1-0.1)	169 (96.5-256.2)	4.8 (2.7-7.3)	4.3 (2.5-6.3)	0.1 (0.1-0.1)	203.6 (117.6-302.9)	5.9 (3.4-8.7)	0.63 (0.43-0.83)	0.97 (0.86-1.08)

Lebanon	3.5 (1.7-6.2)	0.2 (0.1-0.4)	109.3 (58.7-175.5)	5.4 (2.9-8.9)	10.8 (5.8-17.3)	0.2 (0.1-0.3)	288.5 (160.6-446)	4.6 (2.6-7)	-1.07 (-1.23--0.9)	-0.63 (-0.74--0.52)
Lesotho	0.5 (0.2-0.8)	0.1 (0-0.1)	18.8 (10.6-30)	2.4 (1.3-3.9)	0.9 (0.5-1.5)	0.1 (0.1-0.2)	36.7 (20.1-58.8)	3.7 (2-5.9)	2.57 (2.23-2.91)	1.96 (1.78-2.14)
Liberia	0.3 (0.1-0.5)	0 (0-0.1)	15.2 (8.1-24)	1.4 (0.8-2.2)	0.5 (0.2-0.7)	0 (0-0.1)	25.3 (13.7-40.5)	1.2 (0.6-1.9)	-1.05 (-1.16--0.93)	-0.75 (-0.83--0.68)
Libya	0.9 (0.5-1.6)	0.1 (0-0.1)	41.4 (23-64.4)	2.3 (1.3-3.6)	2.4 (1.2-4.4)	0.1 (0-0.1)	122.4 (68.9-192.6)	2.4 (1.3-3.7)	0.97 (0.68-1.26)	0.5 (0.39-0.62)
Lithuania	4.3 (2.3-6.5)	0.1 (0.1-0.2)	207.6 (113.1-313.8)	4.6 (2.5-7)	7.1 (4-10.7)	0.1 (0.1-0.2)	277.6 (160.4-424.9)	5.3 (3-8)	0.33 (0.18-0.48)	0.52 (0.41-0.63)
Luxembourg	1.1 (0.6-1.7)	0.2 (0.1-0.3)	42 (24.8-63.5)	7.8 (4.6-11.8)	1.8 (1-2.8)	0.1 (0.1-0.2)	59.7 (34.3-91.8)	5.6 (3.3-8.7)	-0.72 (-0.94--0.51)	-0.96 (-1.06--0.87)
Madagascar	6 (2.9-9.7)	0.2 (0.1-0.3)	189.5 (99.8-292.7)	4.2 (2.2-6.6)	6 (2.9-9.7)	0.1 (0-0.1)	239.2 (127.1-378.2)	2.2 (1.2-3.5)	-2.33 (-2.73--1.93)	-2.14 (-2.48--1.79)
Malawi	2.3 (1-4.1)	0.1 (0-0.2)	101.8 (53.8-163.5)	3.1 (1.6-5.2)	5.6 (2.7-9.8)	0.1 (0.1-0.2)	231.9 (121.8-367.7)	3.4 (1.8-5.6)	0.18 (0.08-0.27)	0.22 (0.15-0.29)
Malaysia	11.9 (6.7-	0.2	500.2 (281.6-	5.7 (3.1-	32.2 (17.7-	0.2	1283 (758.1-	4.9 (2.9-	-0.09	-0.53

	18.8)	(0.1-0.3)	750.9)	8.6)	49.6)	(0.1-0.3)	1975.6)	7.6)	(-0.5-0.32)	(-0.7--0.35)
Maldives	0.1 (0.1-0.2)	0.3 (0.2-0.6)	5.8 (3.1-8.9)	8.9 (5-14.2)	0.6 (0.3-1)	0.3 (0.1-0.4)	21.2 (11.6-32)	6.9 (3.8-10.3)	-1.22 (-1.33--1.1)	-1.08 (-1.23--0.94)
Mali	0.8 (0.3-1.3)	0 (0-0.1)	39.4 (20.9-64)	1.2 (0.6-1.9)	2.9 (1.4-5)	0.1 (0-0.1)	132.3 (71.1-210.1)	1.8 (1-2.9)	2.08 (1.94-2.21)	1.69 (1.59-1.79)
Malta	0.6 (0.3-0.8)	0.1 (0.1-0.2)	26.1 (14.7-39.2)	6.2 (3.5-9.3)	0.9 (0.5-1.4)	0.1 (0-0.1)	32.6 (18.7-50.1)	3.7 (2.1-5.6)	-1.54 (-1.65--1.43)	-1.35 (-1.58--1.13)
Marshall Islands	0 (0-0)	0.1 (0.1-0.2)	0.9 (0.5-1.5)	5.2 (3-8)	0.1 (0-0.1)	0.2 (0.1-0.3)	2.5 (1.3-3.8)	6.1 (3.4-9.4)	0.61 (0.53-0.68)	0.58 (0.52-0.65)
Mauritania	0.4 (0.2-0.6)	0 (0-0.1)	16.2 (9.1-24.9)	1.7 (0.9-2.6)	0.7 (0.3-1.1)	0 (0-0.1)	29.9 (16.1-47.1)	1.4 (0.8-2.2)	-0.66 (-0.81--0.51)	-0.71 (-0.79--0.64)
Mauritius	0.8 (0.5-1.1)	0.2 (0.1-0.2)	38.1 (22.3-57.5)	5.5 (3.2-8.4)	2.4 (1.4-3.5)	0.1 (0.1-0.2)	91.2 (53.8-138.6)	5.1 (3-7.7)	-0.03 (-0.16-0.11)	-0.31 (-0.45--0.16)
Mexico	69.9 (41.5-102.8)	0.3 (0.1-0.4)	2720.9 (1613.6-4051.3)	7.2 (4.2-10.6)	112.7 (66.5-166.2)	0.1 (0.1-0.1)	4596.9 (2616.1-7140.5)	3.7 (2.1-5.7)	-3.15 (-3.35--2.95)	-2.34 (-2.47--2.22)
Micronesia (Federated States of)	0.1 (0.1-0.2)	0.3 (0.2-	5.7 (3.5-8.5)	11.1 (6.7-	0.2 (0.1-0.3)	0.3 (0.2-	9.7 (5.5-14.4)	11.5 (6.6-	0.02 (0-0.04)	0.11 (0.09-0.13)

		0.5)		16.6)		0.5)		17.1)		
Monaco	0.1 (0.1-0.2)	0.2 (0.1-0.2)	4.8 (2.7-7.7)	7.3 (4.1-11.6)	0.1 (0.1-0.2)	0.1 (0.1-0.2)	4.9 (2.7-7.9)	5.4 (3-8.5)	-0.9 (-1.07--0.73)	-1.09 (-1.16--1.03)
Mongolia	0.6 (0.3-0.9)	0.1 (0-0.1)	41.7 (23-63.4)	3.9 (2.1-6)	1.3 (0.7-1.9)	0.1 (0-0.1)	109.9 (60.8-164.8)	4.5 (2.5-6.8)	0.07 (-0.01-0.14)	0.5 (0.45-0.55)
Montenegro	3 (1.7-4.4)	0.5 (0.3-0.8)	89.6 (53.3-128.7)	14.3 (8.5-20.7)	5.3 (3-8.2)	0.6 (0.3-0.9)	142.6 (83.3-216.5)	14.6 (8.5-22.1)	0.55 (0.44-0.66)	0.12 (-0.05-0.28)
Morocco	5.6 (2.4-9.4)	0 (0-0.1)	250 (130.7-397.1)	1.8 (1-2.9)	10.9 (5.9-16.9)	0 (0-0.1)	502.9 (281.2-773.7)	1.5 (0.8-2.3)	-1.1 (-1.3--0.9)	-0.89 (-1.04--0.74)
Mozambique	3.4 (1.4-5.8)	0.1 (0-0.2)	144.2 (75.4-228.7)	2.6 (1.4-4.2)	7.6 (3.7-12.6)	0.1 (0-0.2)	305.7 (166.3-493.8)	2.8 (1.5-4.6)	0.67 (0.56-0.78)	0.55 (0.45-0.65)
Myanmar	47.8 (25.7-79.4)	0.3 (0.2-0.6)	2094.4 (1176.6-3228.4)	10.6 (6.1-16.5)	79.1 (44.2-121.7)	0.2 (0.1-0.4)	2765.3 (1568.3-4179.2)	6.3 (3.6-9.7)	-1.63 (-1.78--1.47)	-1.95 (-2.07--1.83)
Namibia	0.7 (0.4-1.1)	0.2 (0.1-0.3)	27.6 (16.1-42.5)	5.4 (3-8.5)	1.4 (0.8-2.3)	0.2 (0.1-0.3)	49.1 (27.4-74.8)	4.3 (2.4-6.7)	-0.74 (-1.04--0.45)	-0.96 (-1.16--0.77)
Nauru	0 (0-0)	0.4 (0.2-0.6)	0.7 (0.4-1)	13.2 (7.9-19.1)	0 (0-0)	0.4 (0.2-0.6)	0.8 (0.5-1.2)	12.1 (6.9-17.8)	-0.38 (-0.5--0.25)	-0.37 (-0.5--0.24)

Nepal	11.7 (5.3-20.3)	0.2 (0.1-0.4)	565.9 (318.9-868.8)	7.3 (4.1-11.1)	37.6 (19.3-61.9)	0.2 (0.1-0.4)	1212.9 (678.9-1923.2)	6.1 (3.4-9.8)	0.35 (0.11-0.59)	-0.81 (-0.91--0.71)
Netherlands	68.3 (38.8-102.1)	0.3 (0.2-0.5)	2157 (1263.1-3274)	10.9 (6.4-16.4)	83.8 (47.8-128.1)	0.2 (0.1-0.3)	2425.3 (1387.7-3735.6)	6.8 (3.9-10.5)	-1.54 (-1.6--1.47)	-1.61 (-1.68--1.54)
New Zealand	10 (5.9-14.9)	0.3 (0.2-0.4)	339.7 (196.3-516.5)	8.8 (5.1-13.2)	19.8 (11.2-30.5)	0.2 (0.1-0.3)	573.6 (328.3-875.3)	6.9 (3.9-10.5)	-0.47 (-0.71--0.23)	-0.68 (-0.89--0.46)
Nicaragua	0.9 (0.5-1.4)	0.1 (0-0.1)	49.8 (27.5-76.6)	3.3 (1.8-5.1)	2.8 (1.5-4.4)	0.1 (0-0.1)	143 (80.1-226.7)	2.9 (1.7-4.7)	0.09 (-0.09-0.27)	-0.09 (-0.22-0.04)
Niger	0.4 (0.1-0.7)	0 (0-0.1)	18.7 (9.2-30.7)	0.8 (0.4-1.4)	1.4 (0.5-2.6)	0 (0-0.1)	54.5 (26.5-92.4)	0.9 (0.4-1.5)	0.68 (0.42-0.94)	0 (-0.17-0.17)
Nigeria	12.8 (6.1-21.2)	0 (0-0.1)	425.5 (227.9-666.4)	1.1 (0.6-1.8)	11 (5.3-18.1)	0 (0-0)	527.4 (274.4-833.6)	0.6 (0.3-1)	-3.47 (-3.65--3.28)	-2.18 (-2.32--2.04)
Niue	0 (0-0)	0.2 (0.1-0.2)	0.1 (0.1-0.2)	6 (3.5-8.9)	0 (0-0)	0.2 (0.1-0.2)	0.1 (0.1-0.2)	6 (3.4-9)	-0.27 (-0.34--0.19)	-0.23 (-0.31--0.15)
North Macedonia	3.1 (1.7-4.7)	0.2 (0.1-0.3)	133.1 (76.7-201)	7.2 (4.2-11)	6.4 (3.2-10.8)	0.3 (0.1-0.4)	245.1 (133.2-379.2)	7.8 (4.4-11.8)	0.89 (0.29-1.5)	0.22 (-0.01-0.45)
Northern Mariana	0 (0-0.1)	0.2	1.8 (1.1-2.7)	8.3 (4.8-	0.1 (0.1-0.1)	0.2	4.4 (2.6-6.6)	7.6 (4.4-	-0.28	-0.36

Islands		(0.1-0.3)		12.3)		(0.1-0.3)		11.1)	(-0.36--0.19)	(-0.44--0.28)
Norway	22.2 (12.3-33)	0.3 (0.2-0.4)	692.8 (403.2-1077.9)	10.3 (5.9-15.8)	14.9 (8.1-24.2)	0.1 (0.1-0.2)	464.6 (259.8-750.3)	4.8 (2.7-7.5)	-3.49 (-3.82--3.16)	-3.1 (-3.32--2.89)
Oman	0.4 (0.2-0.6)	0.1 (0-0.1)	14.7 (8.3-23)	2.1 (1.2-3.4)	0.9 (0.5-1.4)	0.1 (0-0.1)	39.3 (21.1-61.8)	1.9 (1-2.9)	0.35 (-0.14-0.85)	-0.03 (-0.36-0.31)
Pakistan	56.9 (26-93.8)	0.1 (0.1-0.2)	2832.4 (1519-4441.5)	5.5 (3-8.7)	105.5 (54.3-166.7)	0.1 (0.1-0.2)	4471.5 (2465.3-6675.8)	4.2 (2.3-6.5)	-0.47 (-0.81--0.14)	-1.1 (-1.31--0.9)
Palau	0 (0-0)	0.1 (0.1-0.2)	0.5 (0.3-0.7)	4.5 (2.5-6.8)	0 (0-0)	0.1 (0-0.1)	1 (0.6-1.5)	4.1 (2.4-6.2)	-0.64 (-0.68--0.59)	-0.38 (-0.45--0.3)
Palestine	1.1 (0.6-1.7)	0.2 (0.1-0.3)	29.4 (16.8-43.9)	3.9 (2.1-5.9)	2.2 (1.2-3.4)	0.1 (0.1-0.2)	77 (44.4-115.3)	3.5 (2-5.3)	-1.27 (-1.52--1.01)	-0.75 (-0.91--0.59)
Panama	1.4 (0.8-2.2)	0.1 (0.1-0.2)	54.5 (31.2-84.4)	3.8 (2.2-5.9)	3.4 (1.8-5.5)	0.1 (0-0.1)	114.4 (61.8-182.1)	2.6 (1.4-4.1)	-1.7 (-1.87--1.53)	-1.58 (-1.71--1.44)
Papua New Guinea	2 (0.9-3.3)	0.1 (0.1-0.2)	111.8 (59.5-174.9)	5.3 (2.8-8.3)	5.7 (2.8-9.2)	0.1 (0.1-0.2)	312.2 (168.5-480.3)	5 (2.7-7.7)	-0.35 (-0.45--0.26)	-0.39 (-0.48--0.31)
Paraguay	6.5 (3.6-9.7)	0.3 (0.2-	232.3 (134.3-355.5)	11 (6.4-16.8)	13.7 (6.7-22.5)	0.3 (0.1-	442.5 (242.6-686.6)	7.9 (4.3-12.3)	-0.85 (-1.02-	-1.26 (-1.37-

		0.5)				0.4)			-0.68)	-1.14)
Peru	8.3 (4.4-12.6)	0.1 (0-0.1)	310.7 (171-474.5)	2.7 (1.5-4)	21.2 (11.4-36)	0.1 (0-0.1)	893.3 (483.5-1389)	2.7 (1.4-4.1)	-1.13 (-1.47--0.79)	-0.27 (-0.45--0.1)
Philippines	46.8 (27.2-72.1)	0.3 (0.2-0.5)	2116.8 (1227.1-3147)	8.6 (5-13.1)	99 (58-151)	0.2 (0.1-0.3)	4320.5 (2585.7-6364.4)	5.6 (3.3-8.4)	-1.73 (-1.97--1.48)	-1.44 (-1.61--1.27)
Poland	97.5 (58.8-140.4)	0.2 (0.1-0.3)	3735.9 (2222.7-5514.9)	8.6 (5.1-12.7)	98.8 (58.6-146.5)	0.1 (0.1-0.2)	4666.2 (2666.1-7227.6)	6.7 (3.9-10.3)	-1.46 (-2.05--0.87)	-0.85 (-1.19--0.51)
Portugal	10.9 (6.5-15.6)	0.1 (0-0.1)	636.7 (355.6-981)	4.7 (2.6-7.2)	13.3 (7.4-20.1)	0 (0-0.1)	658 (377.5-1015.3)	3.1 (1.8-4.8)	-1.75 (-1.93--1.57)	-1.35 (-1.43--1.26)
Puerto Rico	3.9 (2.2-5.9)	0.1 (0.1-0.2)	146.5 (81.7-227.9)	4.2 (2.3-6.5)	6.6 (3.4-10.7)	0.1 (0-0.1)	226.1 (122.8-358.3)	3.3 (1.8-5.1)	-1.89 (-2.04--1.73)	-0.97 (-1.05--0.89)
Qatar	0 (0-0.1)	0.1 (0-0.2)	2.9 (1.6-4.8)	2.4 (1.3-3.9)	0.2 (0.1-0.5)	0 (0-0.1)	22.7 (10.9-40.5)	1.7 (0.9-2.9)	-2.85 (-3.44--2.26)	-1.07 (-1.31--0.83)
Republic of Korea	36 (21-57.6)	0.2 (0.1-0.3)	2147.7 (1229.2-3289.7)	7.2 (4.1-10.9)	129 (67.7-207.7)	0.1 (0.1-0.2)	5102.9 (2945.6-7752.3)	5.7 (3.3-8.6)	-0.65 (-0.78--0.52)	-0.75 (-0.86--0.64)
Republic of Moldova	2.2 (1.3-3.3)	0.1 (0-0.1)	156.8 (86.4-240.5)	3.5 (1.9-5.4)	4.7 (2.8-6.8)	0.1 (0-0.1)	261.9 (154.2-396.3)	4.5 (2.6-6.8)	0.82 (0.61-1.04)	0.9 (0.79-1.02)

Romania	23.2 (12.9-34.6)	0.1 (0.1-0.2)	1340.6 (749-2048.5)	4.9 (2.7-7.5)	31.1 (17.6-46.3)	0.1 (0-0.1)	1240.5 (711-1908.8)	3.5 (2-5.4)	-1.4 (-1.63--1.17)	-1.4 (-1.56--1.24)
Russian Federation	117.8 (66.2-176.4)	0.1 (0-0.1)	7849.8 (4410.9-11862.3)	4.3 (2.4-6.5)	243.8 (149.8-354.8)	0.1 (0.1-0.1)	12985.6 (7782.5-19300.5)	5.5 (3.3-8.3)	1.12 (0.73-1.52)	0.85 (0.52-1.18)
Rwanda	3.8 (1.6-6.8)	0.2 (0.1-0.4)	137.3 (69.1-235.8)	5.8 (2.9-9.7)	6.8 (2.3-13.2)	0.2 (0.1-0.4)	263.7 (128.2-438.2)	5 (2.4-8.8)	-1.13 (-1.41--0.85)	-0.84 (-1.03--0.64)
Saint Kitts and Nevis	0 (0-0.1)	0.1 (0.1-0.2)	1 (0.6-1.6)	2.9 (1.6-4.5)	0 (0-0.1)	0.1 (0-0.1)	1.5 (0.9-2.5)	2.4 (1.3-3.7)	-0.68 (-0.85--0.51)	-0.76 (-0.9--0.63)
Saint Lucia	0.1 (0.1-0.2)	0.2 (0.1-0.2)	3.8 (2.2-5.8)	4.7 (2.7-7.1)	0.2 (0.1-0.4)	0.1 (0.1-0.2)	7.8 (4.3-11.9)	3.2 (1.8-4.9)	-2.28 (-2.58--1.98)	-1.52 (-1.66--1.39)
Saint Vincent and the Grenadines	0.1 (0-0.1)	0.1 (0.1-0.2)	2.6 (1.5-3.9)	3.7 (2.2-5.7)	0.1 (0.1-0.2)	0.1 (0.1-0.2)	4.9 (2.8-7.6)	3.5 (2-5.3)	-0.27 (-0.52--0.02)	-0.2 (-0.35--0.05)
Samoa	0.2 (0.1-0.3)	0.2 (0.1-0.3)	6.9 (4-10.3)	7.9 (4.5-11.7)	0.3 (0.1-0.4)	0.2 (0.1-0.3)	10.7 (6.1-16.2)	7.2 (4.1-10.9)	-0.61 (-0.72--0.5)	-0.55 (-0.65--0.44)
San Marino	0.1 (0-0.1)	0.2 (0.1-0.3)	2.5 (1.5-3.8)	7.3 (4.2-10.9)	0.1 (0-0.1)	0.1 (0-0.1)	2.8 (1.5-4.4)	3.9 (2.1-6.3)	-2.33 (-2.68--1.98)	-1.93 (-2.05--1.81)
Sao Tome and Principe	0 (0-0)	0 (0-0)	0.5 (0.3-0.8)	0.8 (0.4-	0 (0-0)	0 (0-0)	1.1 (0.6-1.7)	1 (0.5-	1.36 (1.1-	0.44

				1.3)				1.5)	1.62)	(0.21-0.68)
Saudi Arabia	1.2 (0.7-2)	0 (0-0)	66.5 (35.9-106)	1.1 (0.6-1.6)	5.2 (2.7-8)	0 (0-0.1)	370.8 (200.8-567.8)	1.5 (0.8-2.3)	0.73 (0.57-0.88)	1.22 (1.11-1.33)
Senegal	0.9 (0.4-1.4)	0 (0-0.1)	48.5 (26.4-74.6)	1.5 (0.8-2.2)	1.7 (0.9-2.7)	0 (0-0)	82.8 (44.8-133.6)	1.1 (0.6-1.7)	-0.88 (-0.99--0.77)	-1.34 (-1.41--1.26)
Serbia	14.9 (8.5-21.9)	0.2 (0.1-0.3)	664.3 (377.2-1003.1)	6.1 (3.5-9.1)	23.3 (12.6-35.5)	0.1 (0.1-0.2)	775.8 (443.9-1177)	4.8 (2.8-7.3)	-1 (-1.16--0.84)	-0.85 (-0.93--0.77)
Seychelles	0.1 (0.1-0.2)	0.2 (0.1-0.3)	3.5 (1.9-5.3)	6.2 (3.5-9.5)	0.1 (0.1-0.2)	0.2 (0.1-0.3)	6 (3.4-9.3)	5.4 (3.1-8.4)	-0.63 (-0.77--0.49)	-0.54 (-0.61--0.48)
Sierra Leone	0.9 (0.4-1.5)	0.1 (0-0.1)	45.2 (24.6-70)	2.3 (1.2-3.6)	1.2 (0.7-2)	0 (0-0.1)	68.3 (36.9-107.3)	1.8 (1-2.9)	-0.85 (-0.89--0.8)	-0.79 (-0.81--0.77)
Singapore	1.2 (0.7-1.8)	0.1 (0-0.1)	78.6 (43.4-122.8)	3.3 (1.9-5.2)	1.9 (1.1-2.9)	0 (0-0)	160.1 (86-255)	1.8 (1-2.9)	-3.51 (-3.74--3.29)	-2.12 (-2.22--2.02)
Slovakia	9.4 (5.5-13.9)	0.2 (0.1-0.2)	455.8 (266.7-685.1)	7.7 (4.5-11.6)	14 (7.7-21.7)	0.1 (0.1-0.2)	645.8 (363.7-957.3)	6.8 (3.9-10.2)	-0.08 (-0.2-0.05)	-0.25 (-0.33--0.17)
Slovenia	1.8 (1-2.6)	0.1 (0-0.1)	97.6 (53.8-149.2)	4 (2.2-6.1)	2.9 (1.6-4.6)	0.1 (0-0.1)	146.7 (82.1-225.6)	3.6 (2-5.5)	-0.16 (-0.7-0.38)	0.05 (-0.21-

										0.31)
Solomon Islands	0.2 (0.1-0.3)	0.2 (0.1-0.3)	10.1 (5.5-16.2)	7.1 (3.8-11.1)	0.6 (0.3-0.9)	0.2 (0.1-0.3)	29.2 (16.6-45.6)	7.7 (4.4-12)	0.47 (0.31-0.63)	0.44 (0.29-0.58)
Somalia	1.1 (0.4-1.9)	0.1 (0-0.1)	54.2 (26.9-90.6)	2.4 (1.2-3.9)	1.7 (0.5-3.6)	0 (0-0.1)	102.5 (48.3-184.3)	1.7 (0.8-2.9)	-1.63 (-1.81--1.45)	-1.14 (-1.29--1)
South Africa	21.1 (12.2-32.3)	0.1 (0.1-0.2)	994.6 (571.6-1476.4)	4.9 (2.8-7.4)	28.8 (16.5-42.9)	0.1 (0-0.1)	1387.1 (820.6-2076.3)	2.9 (1.8-4.4)	-1.98 (-2.21--1.75)	-1.81 (-1.87--1.75)
South Sudan	2 (0.7-3.5)	0.1 (0-0.2)	74.6 (37.4-120.4)	3.1 (1.5-4.9)	1.8 (0.7-3.3)	0.1 (0-0.1)	83.1 (42.6-136.7)	2.2 (1.1-3.7)	-1.26 (-1.43--1.1)	-1.13 (-1.29--0.98)
Spain	85 (51.2-125)	0.2 (0.1-0.2)	4152.1 (2439.3-6327.9)	7.9 (4.6-12.1)	103.8 (58.5-157.4)	0.1 (0-0.1)	4787.1 (2789.1-7286.6)	5.3 (3.1-8.2)	-1.92 (-2.04--1.8)	-1.12 (-1.35--0.89)
Sri Lanka	9.8 (5.4-15.2)	0.2 (0.1-0.3)	495.5 (278.3-767.6)	5.3 (3-8.2)	17 (8.2-28.8)	0.1 (0-0.1)	757 (414-1140.9)	3 (1.6-4.5)	-1.63 (-1.87--1.38)	-1.82 (-1.97--1.68)
Sudan	3.4 (1.5-6.2)	0.1 (0-0.1)	150.7 (83.9-240.4)	1.8 (1-2.9)	7.2 (3.8-11.8)	0.1 (0-0.1)	301.9 (162.6-478.5)	1.7 (0.9-2.7)	-0.18 (-0.22--0.14)	-0.27 (-0.34--0.19)
Suriname	0.4 (0.2-0.5)	0.2 (0.1-0.2)	15.8 (9.3-23.8)	6.1 (3.6-9.2)	0.7 (0.4-1.1)	0.1 (0.1-0.2)	28.8 (16.5-43.8)	4.5 (2.5-6.8)	-1.22 (-1.43--1.01)	-1.19 (-1.35--1.03)

Sweden	28.9 (16.2-45.5)	0.2 (0.1-0.3)	1148.9 (642.4-1799.5)	7.6 (4.4-11.8)	67.4 (35.6-107.6)	0.2 (0.1-0.4)	2056.5 (1109.1-3233.7)	9.3 (5-14.6)	1.49 (1.14-1.83)	0.6 (0.43-0.77)
Switzerland	12.1 (6.9-18.7)	0.1 (0.1-0.2)	453 (256.9-700.7)	4.4 (2.5-6.7)	24.2 (13.5-37.3)	0.1 (0.1-0.2)	683.2 (389.6-1061.4)	3.7 (2.1-5.7)	0.31 (0.14-0.49)	-0.13 (-0.4-0.13)
Syrian Arab Republic	5.5 (2.7-8.7)	0.1 (0.1-0.2)	184.5 (100.8-285.8)	3.9 (2.1-6.1)	9.7 (5.1-15.7)	0.1 (0.1-0.2)	382.6 (211.1-584.5)	3.3 (1.8-5.1)	-0.75 (-1.01--0.48)	-0.8 (-0.93--0.67)
Taiwan (Province of China)	17.5 (11-25)	0.2 (0.1-0.3)	915.3 (534.1-1366.5)	6.3 (3.7-9.4)	58.9 (32.2-86.1)	0.1 (0.1-0.2)	1978.4 (1088.1-2935.6)	4.6 (2.6-6.9)	-1.38 (-1.8--0.95)	-1.21 (-1.44--0.99)
Tajikistan	0.8 (0.4-1.4)	0 (0-0.1)	69.7 (38.3-109.4)	2.5 (1.4-4)	0.8 (0.4-1.2)	0 (0-0)	95.2 (48-151.9)	1.6 (0.8-2.5)	-2.32 (-2.62--2.02)	-1.55 (-1.66--1.44)
Thailand	66 (35.1-104.6)	0.3 (0.2-0.5)	2360.6 (1385.7-3583.5)	7.6 (4.4-11.6)	167.9 (88.8-265.4)	0.2 (0.1-0.2)	5208.7 (2975.7-7745.4)	4.8 (2.7-7.1)	-2.78 (-2.98--2.57)	-1.87 (-1.98--1.76)
Timor-Leste	0.2 (0.1-0.4)	0.2 (0.1-0.3)	14.9 (8.3-22.7)	5.9 (3.2-8.9)	1 (0.5-1.5)	0.2 (0.1-0.3)	44.7 (24.4-68.5)	5.9 (3.3-9)	0.52 (0.46-0.58)	-0.03 (-0.07-0.01)
Togo	0.6 (0.3-1)	0.1 (0-0.1)	26.5 (14.5-41.4)	2.5 (1.3-3.9)	1.3 (0.7-2.1)	0.1 (0-0.1)	60.8 (33.3-96.6)	1.7 (0.9-2.7)	-1.49 (-1.57--1.4)	-1.33 (-1.38--1.27)
Tokelau	0 (0-0)	0.2	0.1 (0.1-0.1)	7.4 (4.2-	0 (0-0)	0.2	0.1 (0.1-0.2)	6.8 (3.8-	-0.65	-0.45

		(0.1-0.3)		11.2)		(0.1-0.3)		10.6)	(-0.73--0.56)	(-0.51--0.38)
Tonga	0.1 (0.1-0.2)	0.2 (0.1-0.4)	4.6 (2.7-6.8)	8.5 (5-12.8)	0.2 (0.1-0.3)	0.2 (0.1-0.3)	6.1 (3.4-9.3)	7.7 (4.2-11.6)	-0.47 (-0.59--0.35)	-0.46 (-0.53--0.38)
Trinidad and Tobago	0.9 (0.6-1.4)	0.1 (0.1-0.2)	42.8 (23.3-66.1)	5.2 (2.9-8.1)	1.9 (1-2.9)	0.1 (0.1-0.2)	81.2 (43.9-124.8)	4.2 (2.3-6.5)	-1.13 (-1.25--1.01)	-0.79 (-0.87--0.71)
Tunisia	4.5 (2.5-7.4)	0.1 (0.1-0.2)	167.9 (94.7-250.1)	3.9 (2.2-6)	17.1 (8.5-27.9)	0.2 (0.1-0.3)	470.1 (259.5-713.9)	3.9 (2.1-5.9)	0.09 (-0.09-0.28)	-0.21 (-0.3--0.12)
Turkey	31.1 (17-50.2)	0.1 (0.1-0.2)	1261.9 (718.2-1984.6)	3.7 (2.1-5.8)	64.8 (36.4-100.5)	0.1 (0-0.1)	2325 (1321-3446.3)	2.5 (1.4-3.7)	-1.38 (-1.74--1.01)	-1.61 (-1.84--1.38)
Turkmenistan	0.8 (0.5-1.2)	0 (0-0.1)	55.9 (31.7-85.7)	2.9 (1.7-4.4)	1.6 (0.9-2.3)	0 (0-0.1)	99.8 (55.4-155.9)	2.4 (1.3-3.6)	-0.6 (-0.78--0.42)	-0.73 (-0.92--0.53)
Tuvalu	0 (0-0)	0.2 (0.1-0.3)	0.6 (0.3-0.9)	8 (4.4-12.4)	0 (0-0)	0.2 (0.1-0.3)	0.9 (0.5-1.3)	8.3 (4.9-12.3)	0.02 (-0.02-0.07)	0.1 (0.06-0.14)
Uganda	3 (1.2-5.3)	0.1 (0-0.1)	129.4 (64.7-208)	2.2 (1.1-3.5)	5.6 (2.5-10.3)	0.1 (0-0.1)	255.4 (133.8-422.2)	1.8 (0.9-3)	-1.28 (-1.51--1.05)	-1.15 (-1.38--0.92)
Ukraine	53.4 (30.9-80.6)	0.1 (0-0.1)	3139.4 (1779.2-4740.6)	4.5 (2.5-6.7)	62.6 (33.9-97.4)	0.1 (0-0.1)	3007.4 (1681.4-4663.7)	4 (2.3-6.3)	-0.56 (-0.98-	-0.49 (-0.78-

									-0.14)	-0.19)
United Arab Emirates	0.2 (0.1-0.3)	0.1 (0-0.1)	12.3 (6.7-20.5)	2.3 (1.3-3.6)	0.8 (0.5-1.4)	0.1 (0-0.1)	100.9 (48.6-168.7)	2.1 (1.2-3.4)	1.69 (0.72-2.67)	0.75 (0.3-1.2)
United Kingdom	267.2 (162.3-390.9)	0.3 (0.2-0.4)	7936.6 (4610.9-12057.5)	8.7 (5.1-13.2)	278.5 (157.4-441.4)	0.2 (0.1-0.3)	7377.1 (4184.2-11287.9)	5.6 (3.2-8.7)	-1.38 (-1.53--1.23)	-1.52 (-1.64--1.39)
United Republic of Tanzania	11.5 (4.9-19.3)	0.2 (0.1-0.3)	438.4 (225.8-701.9)	4.5 (2.4-7.1)	19.1 (8.5-34.2)	0.1 (0-0.2)	792.1 (402.3-1297.3)	3.2 (1.6-5.3)	-1.93 (-2.13--1.72)	-1.47 (-1.64--1.31)
United States of America	466.3 (275.7-697.8)	0.1 (0.1-0.2)	21925.2 (12394.6-34060.4)	7 (4-10.8)	939.7 (536.4-1438.7)	0.1 (0.1-0.2)	36860.5 (21247.2-55370.3)	6.4 (3.7-9.5)	0.1 (0.02-0.18)	-0.31 (-0.36--0.25)
United States Virgin Islands	0.1 (0-0.1)	0.1 (0.1-0.2)	2.9 (1.6-4.6)	3.5 (1.9-5.5)	0.1 (0.1-0.2)	0.1 (0-0.1)	4.2 (2.3-6.7)	2.4 (1.3-3.7)	-1.86 (-2.2--1.51)	-1.27 (-1.46--1.08)
Uruguay	2.7 (1.6-3.9)	0.1 (0-0.1)	144.7 (79.8-219.6)	3.8 (2.1-5.7)	5.1 (2.9-7.9)	0.1 (0-0.1)	188.3 (107.3-288.6)	3.6 (2.1-5.5)	0.6 (0.42-0.77)	-0.27 (-0.39--0.16)
Uzbekistan	1.4 (0.6-2.7)	0 (0-0)	154.1 (83.3-246.4)	1.3 (0.7-2.1)	6 (3.4-8.8)	0 (0-0)	590.5 (312.6-912.7)	2.1 (1.1-3.2)	2.75 (2.48-3.02)	1.58 (1.3-1.87)
Vanuatu	0.1 (0-0.1)	0.1 (0.1-0.2)	4 (2.2-6.5)	6 (3.3-9.7)	0.2 (0.1-0.3)	0.1 (0.1-0.2)	9.3 (5.2-14.3)	4.9 (2.7-7.7)	-0.84 (-0.98--0.71)	-0.89 (-1.02--0.77)

Venezuela (Bolivarian Republic of)	10.9 (6.2-16.2)	0.1 (0.1-0.2)	525.2 (291.9-789.1)	5.6 (3.2-8.6)	24.6 (13.3-38.8)	0.1 (0-0.1)	1085.8 (604.3-1682.3)	3.6 (2-5.7)	-1.7 (-1.9--1.51)	-1.56 (-1.65--1.47)
Viet Nam	56.6 (31.6-91.5)	0.2 (0.1-0.3)	2300.5 (1348.1-3489.4)	6.1 (3.6-9.2)	136.7 (77.5-217.1)	0.2 (0.1-0.3)	5615.7 (3259.5-8274)	5.8 (3.4-8.7)	-0.53 (-0.74--0.32)	-0.42 (-0.52--0.32)
Yemen	2.7 (1.2-4.6)	0.1 (0-0.1)	122.5 (68.1-192.7)	2.7 (1.5-4.2)	9.9 (4.7-17.1)	0.1 (0-0.2)	392.4 (213-639.8)	3 (1.6-4.9)	0.89 (0.83-0.95)	0.44 (0.4-0.47)
Zambia	2.5 (1.3-4)	0.1 (0.1-0.2)	93.4 (52.6-143.7)	3.7 (2.1-5.8)	7.5 (3-14.9)	0.2 (0.1-0.3)	252.5 (111.3-463.6)	4 (1.8-7.2)	0.56 (0.27-0.85)	0.17 (0-0.35)
Zimbabwe	2.8 (1.6-4.3)	0.1 (0.1-0.2)	134.3 (77.8-199)	3.8 (2.1-5.7)	5 (2.7-8)	0.1 (0.1-0.2)	235.2 (134.6-358.5)	3.7 (2.1-5.7)	0.31 (-0.05-0.68)	0.12 (-0.09-0.33)

*The all-age mortality is equivalent to the crude mortality rate.

*The numbers in parentheses in the table represent the 95% confidence interval.

Abbreviation: AF/AFL, atrial fibrillation and atrial flutter; ASMR, age-standardized mortality rate; DALYs, disability-adjusted life years; ASDR, age standardized DALYs rates; EAPCs estimated annual percentage changes.

Table S2. Joinpoint regression analysis of ASMR and ASDR of AF/AFL attributable to tobacco at the global level, 1990–2021.

Measure	Years	APC (95% CI)	AAPC (95% CI)	Joinpoints
Deaths	1990–1996	+0.18*		1996, 2004, 2007
	1997–2003	-0.73*	-0.66*	
	2004–2006	-1.38*		
	2007–2021	-0.83*		
DALYs	1990–1995	-0.52*	-0.72*	1995, 2002, 2005, 2012, 2015
	1996–2000	-0.71*		
	2002–2004	-0.39		
	2005–2010	-0.87*		
	2012–2013	-1.11*		
	2015–2021	-0.68*		

Abbreviations: AF/AFL, atrial fibrillation and atrial flutter; DALYs, disability-adjusted life years; ASMR, age-standardized mortality rate; ASDR, age standardized DALYs rates; APC, annual percentage change; AAPC, average annual percentage change ； * Indicates statistically significant APC ($p < 0.05$).

© 2026 Chen L. et al.
